# Parallel Multifactorial Process Optimization and Intensification for High-Yield Production of Live YF17D-Vectored Zika Vaccine

**DOI:** 10.3390/vaccines12070755

**Published:** 2024-07-09

**Authors:** Sven Göbel, Ozeir Kazemi, Ji Ma, Ingo Jordan, Volker Sandig, Jasmine Paulissen, Winnie Kerstens, Hendrik Jan Thibaut, Udo Reichl, Kai Dallmeier, Yvonne Genzel

**Affiliations:** 1 Bioprocess Engineering, Max Planck Institute for Dynamics of Complex Technical Systems, Sandtorstr. 1, 39106 Magdeburg, Germany; goebel@mpi-magdeburg.mpg.de (S.G.); 2 KU Leuven Department of Microbiology, Immunology & Transplantation, Rega Institute, Molecular Vaccinology and Vaccine Discovery (MVVD), 3000 Leuven, Belgium; kai.dallmeier@kuleuven.be (K.D.); 3 ProBioGen AG, 13086 Berlin, Germany; ingo.jordan@probiogen.de (I.J.); 4 KU Leuven Department of Microbiology, Immunology & Transplantation, Rega Institute, Translational Platform Virology and Chemotherapy (TPVC), 3000 Leuven, Belgium; 5 Bioprocess Engineering, Otto-von-Guericke University, Universitätsplatz 2, 39106 Magdeburg, Germany

**Keywords:** live-attenuated vaccine, process intensification, virus yield, high cell density, suspension cells, YF17D, Zika vaccine

## Abstract

The live-attenuated yellow fever 17D strain is a potent vaccine and viral vector. Its manufacture is based on embryonated chicken eggs or adherent Vero cells. Both processes are unsuitable for rapid and scalable supply. Here, we introduce a high-throughput workflow to identify suspension cells that are fit for the high-yield production of live YF17D-based vaccines in an intensified upstream process. The use of an automated parallel ambr15 microbioreactor system for screening and process optimization has led to the identification of two promising cell lines (AGE1.CR.pIX and HEK_Dyn_) and the establishment of optimized production conditions, which have resulted in a >100-fold increase in virus titers compared to the current state of the art using adherent Vero cells. The process can readily be scaled up from the microbioreactor scale (15 mL) to 1 L stirred tank bioreactors. The viruses produced are genetically stable and maintain their favorable safety and immunogenicity profile, as demonstrated by the absence of neurovirulence in suckling BALB/c mice and consistent seroprotection in AG129 mice. In conclusion, the presented workflow allows for the rapid establishment of a robust, scalable, and high-yield process for the production of live-attenuated orthoflavivirus vaccines, which outperforms current standards. The approach described here can serve as a model for the development of scalable processes and the optimization of yields for other virus-based vaccines that face challenges in meeting growing demands.

## 1. Introduction

The Zika virus (ZIKV) and yellow fever virus (YFV) are enveloped, single-stranded RNA viruses within the *Orthoflavivirus* genus. Both viruses are primarily transmitted by *Aedes* mosquitoes and cause widespread infections in more than 80 countries, particularly in the Americas, Africa [1,2], and, for ZIKV, Asia (Indian subcontinent and Thailand) [3,4]. Due to the occurrence of severe congenital malformations in fetuses [5] and neurological disorders such as Guillain–Barré Syndrome [6] in adults following recent ZIKV infections, the WHO has declared ZIKV a global health emergency. Vaccination would provide a cost-effective and efficient prevention strategy, but currently, licensed vaccines are only available against *Orthoflavivirus* infections with the YFV (i.e., live-attenuated YF17D vaccine), the Japanese encephalitis virus (JEV), the four dengue viruses, and the tick-borne encephalitis virus. Currently, there are more than 50 ZIKV vaccine candidates in various stages of research and development [7], including inactivated [8], subunit [9], live virus [10], viral vector [11,12], and DNA or RNA [13,14] vaccines, with some promising results in pre-clinical and clinical trials [13]. Inactivated and subunit vaccines, while safe, often necessitate the use of high antigen doses and periodic boosting [15]. Live-attenuated vaccines such as YF17D, conversely, typically induce a more comprehensive and lasting immune response. While emerging vaccine technologies such as mRNA vaccines have demonstrated high efficacy in preventing symptomatic COVID-19 infection and disease [16], dependence on ultra-low temperatures for storage and transport, limited production capacity, high cost, and the need for repeated booster dosing hinder widespread use in regions with limited medical infrastructure. As ZIKV is primarily endemic in developing regions in the Southern Hemisphere, the practical significance of a low-cost and single-dose vaccine that does not require specialized cold-chain storage would be advantageous.

The YF17D vaccine is renowned for its exceptional efficacy and safety profile. Over 1 billion doses have been administered globally since its development in the 1930s [17], and the vaccine is associated with a very low incidence of serious adverse side effects (~1 per million [18]). A single dose of YF17D induces well-balanced and durable immunity [19]. By inserting foreign antigens into the viral polyprotein [20], YF17D can also be further used as a platform to engineer novel vaccines against unmet medical needs [12,21,22,23,24,25]. This approach has undergone extensive validation, exemplified by the approval of two human YF17D-based chimeric vaccines targeting Japanese encephalitis virus (Imojev^®^) and dengue virus (Dengvaxia^®^). Nevertheless, many aspects of contemporary vaccinology remain empirical and confined to conventional methodology, including means of manufacture. Realistically, “one-size-fits-all” manufacturing solutions do not exist. However, the exploration of new production platforms and the use of advanced process technologies could open new avenues as seen in the development of first-generation COVID-19 vaccines. Alternative manufacturing modes, besides egg-based production and production in anchorage-dependent cell lines, such as adherent Vero (African green monkey kidney) cells, need to be investigated to satisfy the increasing vaccine demand [26] and to enable rapid, sustainable, robust, and scalable manufacturing of novel vaccine candidates based on the YF17D platform.

In the present study, we have established a workflow that allows for efficient process optimizations by screening multiple parameters in a parallel high-throughput manner using our chimeric YF17D-based Zika virus vaccine candidate YF-ZIK [12,27] as an example. Ultimately, this results in the definition of reliable and efficient process parameters for the establishment of an upstream process for the production of live orthoflavivirus vaccines, with yields that far exceed the current state of the art based on a single cell line. Starting from 14 potential cell lines grown in suspension format, we rapidly identified host cell candidates for subsequent process optimization using a fully automated single-use ambr15 microbioreactor system (Sartorius). Commercially relevant infectious virus titers were obtained with two suspension cell lines (AGE1.CR.pIX and HEK_Dyn_) in batch and fed-batch mode, exceeding the productivity of adherent Vero cells by more than 100-fold at different scales (15 mL to 1 L). Finally, the produced YF-ZIK maintained the expected high genetic stability, favorable safety, and immunogenicity as assessed by sequence analysis and plaque assay in vitro, and in suckling BALB/c mice and AG129 mice. Our approach of rapid process intensification for the production of live orthoflavivirus vaccines should be translatable to a broader spectrum of live viral vaccines.

## 2. Materials and Methods

### 2.1. Cell Lines, Media, and Viral Seed Stock

Detailed information on all used cell lines (e.g., origin, media, and supplier) is provided in Table S1 in the Supplementary file. All suspension cell line cultures (Supplementary Table S1) were cultivated in a Multitron Pro incubator (Infors AG, Bottmingen, Switzerland) at their respective rpms (50 mm orbital throw, Supplementary Table S2) using non-baffled or baffled shake flasks (Corning, Corning, NY, USA, Supplementary Table S2) with a 50 mL working volume (wv) of their specific media (Supplementary Table S1). Cells were seeded at viable cell concentrations (VCCs) between 5.0 and 8.0 × 10^5^ cells/mL and passaged twice a week. Adherent cells (Supplementary Table S1) were cultivated at 37 °C in a 5% CO_2_ atmosphere in T175 cell culture flasks (Greiner, Pleidelsheim, Germany) or 490 cm^2^ roller bottles (Greiner, Germany) with an initial VCC of 3.0–5.0 × 10^5^ cells/mL and a working volume (wv) of 50 mL or 200 mL, respectively. For cell passaging, cells were detached by washing twice with PBS followed by incubation with 1× trypsin/EDTA solution for 10 min. Cell diameter, VCC, and percentage of viability were measured using an automated Vi-CELL XR cell counter (Beckman Coulter, Brea, CA, USA).

The construction and rescue of YF-ZIKprM/E virus (YF-ZIK), a derivative of the YF17D vaccine strain expressing an Asian-lineage ZIKV prM/E envelope, has been described previously [12,27]. After 3 consecutive passages of YF-ZIK in dividing cells, viral seed stocks were prepared. Here, adherent Vero E6 cells were seeded into T175 flasks at 0.5 × 10^5^ cells/mL in 50 mL GMEM-Z medium. After 24 h of growth, the cells in one flask were detached to determine the VCC. The other flasks were washed twice with PBS, and cells were infected at a multiplicity of infection (MOI) of 10^−4^ using 10 mL serum-free VPSFM infection medium containing YF-ZIK. Following a 4 h incubation period, the infection medium was removed, the flasks were washed twice with PBS, and 40 mL of fresh VPSFM was added. The viral stock material was harvested at 5 dpi, centrifuged, and stored at −80 °C until use (7.4 × 10^6^ PFU/mL, passage 4).

### 2.2. YF-ZIK Batch Production

For batch infection experiments, cells were seeded at 8.0 × 10^5^ cells/mL in either an ambr15 vessel (Sartorius, Göttingen, Germany) or 1 L stirred DASGIP bioreactors (STR; Eppendorf AG, Hamburg, Germany) with a starting wv of 15 mL or 350 mL, respectively. Dissolved oxygen was controlled at 50% saturation by sparging of an air–oxygen mixture through a pipe sparger (ambr15) or drilled hole L-sparger (1 L STR). The addition of 1 M sodium bicarbonate (NaHCO_3_) or CO_2_ enrichment were used to control pH. Optionally, antifoam C (3% stock solution) was added to prevent foaming. During cell growth, culture temperature was maintained at 37 °C and pH value was controlled at 7.2 for all cell lines. Ambr15 agitation rates (Supplementary Table S2) were scaled down based on tip speeds described in the literature for 1 L STR runs and kept constant across scaling. For infection in the ambr15 system, the wv was decreased by half and filled up with fresh medium containing YF-ZIK, resulting in a 1.7-fold dilution. For the 1 L STR, the wv was directly increased by the addition of 350 mL fresh medium containing YF-ZIK to achieve a 1:2 dilution. Cell lines were infected at MOIs ranging from 0.1 to 10^−4^. After infection, the cultivation temperature was either maintained at 37 °C or lowered to 32–34 °C. Ambr15 vessels and 1 L STRs were sampled daily to measure VCC, offline pH, and virus titer.

### 2.3. YF-ZIK Fed-Batch Production

Seeding and process control strategies for fed-batch experiments in the ambr15 system were identical to those for batch experiments. Cells were already infected one day after inoculation at an MOI of 10^−3^ for both HEK_Dyn_ and AGE1.CR.pIX (abbreviated as pIX for the remainder of the text) cells. Feeds used for fed-batch mode were the respective media (Dynamis and CD-U7; FB1), CHO Feed 1 (FB2; Sigma Aldrich, St. Louis, MO, USA), or HEKFS (FB3; Sartorius, Germany). The daily volume of concentrated feeds varied from 3 to 10% (*v*/*v*) of the current culture volume and was derived from the manufacturer’s instructions. In total, 33% (*v*/*v*) of total culture volume was added for FB3, 18% (*v*/*v*) for FB2, and 20% (*v*/*v*) for FB1.

### 2.4. Plaque Assay

Infectious YF-ZIK titers were determined by a plaque assay with a coefficient of variance of 25% (±0.15 log). Here, porcine stable kidney (PS) cells (courtesy of A. Teichmann, Robert Koch Institute, Berlin, Germany) were seeded in 24-well plates at a concentration of 0.2 × 10^5^ cells/well. After 2 days of growth, the medium was removed, and diluted virus samples were added and incubated for 4 h at 37 °C. Subsequently, the mixture was overlaid with 1.6% (*w/v*) carboxyl-methyl-cellulose (CMC) in Z-Medium and incubated for four days at 37 °C in 5% CO_2_. After removing the supernatant from each well, the infectious virus was inactivated and the cells were fixed by adding glyoxal solution (197.3 mL 96% ethanol, 78.3 mL 40% glyocal, 7.5 mL acetic acid, filled to 1 L with ddH_2_O) for 15 min and stained with napthalin black (1 g naphthol blue black, 13.6 g sodium acetate, 60 mL glacial acetic acid, added to 1 L ddH_2_O) for 1 h (adapted from [28]). Plaques were manually counted and reported as PFU/mL.

To determine infectious virus input for mice experiments and to analyze plaque phenotypes, selected samples were re-measured with an alternative plaque assay using BHK-21J cells, exactly as described elsewhere [12], with the only modification of a 2 h incubation period at 37 °C (instead of 1 h at room temperature) for virus absorption prior to removal of the inoculum and agar overlay.

### 2.5. Reverse-Transcription Chain Reaction (RT-PCR) and Sequencing

Final harvest samples produced in CR, pIX, HEK_Dyn_, and HEK_FS_ cells in 1 L STRs were subjected to whole-genome sequencing. Using QIAamp Viral RNA Kits (Qiagen, Hilden, Germany), viral RNA was extracted as previously described [12,29]. Subsequently, cDNA fragments spanning the complete genome were synthesized from the extracted RNA using the qScript^TM^ one-step RT-PCR (Quanta bioscience, Beverly, MA, USA) with Kapa Hifi DNA polymerase (Kapa Biosystems, London, UK) according to the manufacturer’s instructions. RT-PCR amplicons were generated using the primers and amplification strategy listed in Supplementary Table S3. These amplicons were designed to cover the entire viral genome with overlapping regions, with each amplicon ranging in size from 1.8 to 3 kb and having a 250–500 bp overlap. For single amplicons, the RT-PCR products were purified using an affinity column purification kit QIAquick Gel Extraction Kit (Qiagen, Germantown, MD, USA) with 30–50 µL of elution buffer. Subsequently, 20 μL of the purified product was mixed with 2 μL of corresponding primers, and the resulting mixture was subjected to Sanger sequencing (Macrogen, Amsterdam, The Netherlands). Final de novo assembly of the viral genome was carried out using Snapgene v6.2 (GSL Biotech, San Diego, CA, USA).

### 2.6. Mice

Pregnant BALB/c dams were purchased from Janvier Labs (Le Genest-Saint-Isle, France). After delivery, to evaluate neurovirulence, 3–6-day-old pups (n = 6–11 per group) were intracranially (i.c.) inoculated with 10 PFU/10 µL or 1000 PFU/10 µL of YF-ZIK YF-ZIK grown on HEK_Dyn_ or pIX cells, 10 PFU/10 µL YF17D (positive control), or medium only (sham; negative control). All pups were monitored daily for morbidity and mortality for 3 weeks after inoculation, as described previously [12].

AG129 mice (Interferon-α/β and γ receptor-deficient; B&K Universal, Marshall Bio resources, Hull, UK) were bred in-house at the University of Leuven (Experimental Animal Facilities) and randomly assigned for this study. The standards outlined by the Federation of European Laboratory Animal Science Associations and Belgian guidelines for animal experimentation were rigorously followed for all mouse experiments. Ethical approval for all experiments was obtained from the Ethical Committee of the Animal Research Center at the University of Leuven (project number P100/2019). Male and female mice (n = 5 per group) aged 6–8 weeks were either sham vaccinated (MEM with 2% FBS) or vaccinated intraperitoneally (i.p.) with 100 PFU/200 µL and 1 × 10^4^ PFU/200 µL YF-ZIK grown on either HEK_Dyn_ or pIX cells, or 270 PFU/200 µL and 2.7 × 10^4^ PFU/200 µL of YF-ZIK grown on Vero E6 cells. Vaccinated animals were bled on days 7, 14, 21, and 28 for determination of serum antibodies.

### 2.7. Serum Neutralizing Titers

Zika (ZIKV-mCherry) reporter virus construction and the serum neutralizing titer (SNT) assay have been described in detail previously [22]. Briefly, serum dilutions were incubated with 10 TCID_50_ ZIKA-mCherry virus in 96-well plates for 1 h at 37 °C. Following this, serum–virus complexes were transferred to 1.0 × 10^4^ BHK-21J cells grown in 96-well plates and incubated for 72 h. The percentage of mCherry-expressing cells was quantified using a Cell Insight CX5/7 High Content Screening platform (ThermoFisher Scientific, Waltham, MA, USA). Neutralization half-maximal inhibitory concentration values were determined by fitting the serum neutralization dilution curve, which was normalized to a virus (100%) and cell control (0%), using Graphpad Prism (GraphPad Software V9.0).

### 2.8. Calculations

Cell-specific growth rate (µ) and cell-specific virus yield (CSVY) were determined using the following equations:

(1) $$µ=\frac{\ln\left({VCC(t}_{n+1}\right)/{VCC(t}_{n}))}{t_{n+1}-t_{n}}$$

(2) $$CSVY=\frac{{PFU}_{max}}{{VCC}_{max}}$$

Here, *VCC* represents the viable cell concentration (cells/mL), *VCC_max_* the maximum viable cell concentration post infection (cells/mL), *t* cultivation time (h), *n* sampling time point (h), and *PFU_max_* the maximum infectious virus titer (PFU).

### 2.9. Statistical Analysis

All statistical evaluations were performed using GraphPad Prism V9 (GraphPad Software, San Diego, CA, USA). The data are presented as either mean values ± standard error of the mean (SEM) or ±standard deviation (STD), as indicated in the figure or table legend. Statistical significance was assessed using a two-way ANOVA followed by Šidák’s multiple comparison test or Kruskal–Wallis test, unless otherwise stated. The log-rank (Mantel–Cox) test was employed to compare survival between groups. Statistical differences between groups are denoted by *p*-values < 0.05: * *p*-values  <  0.05, ** *p*-values  <  0.01, *** *p*-values  <  0.001, **** *p*-values  <  0.0001.

## 3. Results

### 3.1. Identification of High-Producing Host Cell Lines

As a first step of process development, we evaluated 14 suspension cell lines (some growing in varying media, Supplementary Table S1) in parallel using the ambr15 system in various GMP-compliant media for efficient YF-ZIK production (a total of 18 variations, all infected at about 2 × 10^6^ cells/mL, MOI 0.05). Productivity was evaluated based on CSVYs and infectious virus titers in the supernatant and compared to an optimized production in adherent Vero cells (Table 1).

**Figure 1:**
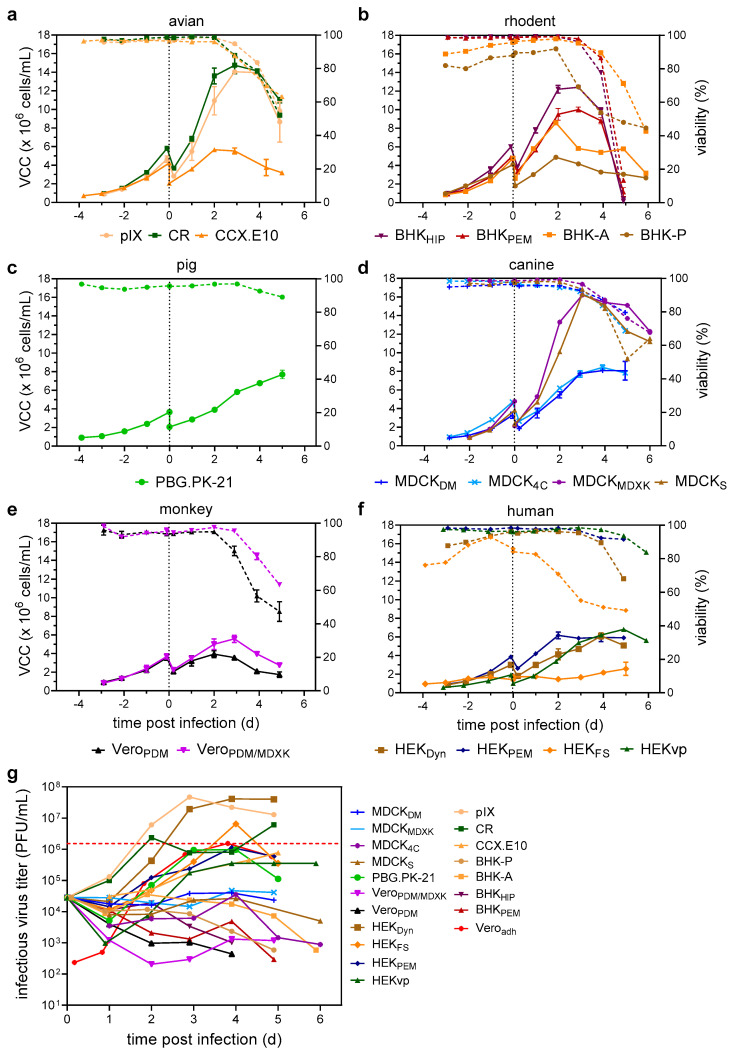
Screening of suspension cell lines for the production of YF-ZIK. Cell and virus growth kinetics in an ambr15 system. (**a**–**g**) VCC (full lines) and culture viabilities (dashed lines) are shown for cell lines grouped by host origin. (**a**) Avian origin: pIX (beige circles), CR (dark green squares), CCX.E10 (orange triangles). (**b**) Rodent origin: BHK_HIP_ (purple triangles), BHK_PEM_ (bordeaux triangles), BHK-A (orange squares), BHK-P (brown circle). (**c**) Pig origin: PBG.PK-21 (green circle). (**d**) Canine origin: MDCK_MDXK_ (purple circle), MDCK_4C_ (blue crosses), MDCK_S_ (brown triangles), MDCK_DM_ (blue dashes). (**e**) Monkey origin: Vero_PDM_ (black triangles), Vero_PDM/MDXK_ (purple triangles). (**f**) Human origin: HEK_FS_ (orange diamond), HEK_Dyn_ (brown square), HEKvp (green triangle), HEK_PEM_ (blue diamonds). (**g**) Infectious virus titers determined by a plaque assay compared to production using Vero_adh_ cells (red diamonds). Dashed black line indicates time of infection; red dashed line maximum infectious virus titer reached using Vero_adh_ cells. Values represented as mean ± STD of two biological replicates.

During 72–96 h of initial cell growth, all but one of the cell lines displayed similar growth characteristics with high viabilities above 95%, achieving µ values from 0.014 to 0.035 1/h (exception: poorly growing HEK_FS_). However, following infection, the cells started to display different cell growth characteristics. While most cell lines continued to grow to VCCs between 4.8 and 10.0 × 10^6^ cells/mL, some such as pIX, CR, MDCK_s_, and MDCK_MDXK_ reached VCCs above 14 × 10^6^ cells/mL (Table 1, Figure 1a–f). Cell-specific growth rates of all infected cell lines were reduced compared to uninfected growth (Table 1). After maximum VCCs (VCC_max_) were reached, viabilities declined rapidly, resulting in final culture viabilities between 50 and 80%, depending on the cell line (Figure 1a–f).

In general, a prolonged growth phase after infection resulted in higher culture viabilities at later stages of infection (e.g., CCX.E10, MDCK_MDXK_, MDCK_DM_, HEKvp) (Figure 1a–f). Plaque assay was used to determine the number of infectious virus particles in the supernatants. Cell lines could be categorized into three groups: high producers, low producers, and no producers (Table 1). Surprisingly, when grown in suspension, Vero, MDCK, and BHK cells showed no evidence of virus replication, regardless of the used media or source. Here, the infectious virus titer either remained stable (at the calculated input titer corresponding to an MOI of 0.05) or decreased over time (Figure 1g). Low producers, including CCX.E10, HEKvp, and PBG.PK-21, were found to be susceptible to YF-ZIK, yet viral titers in the supernatant reached only low levels (1.0–8.0 × 10^5^ PFU/mL). The highest infectious virus titers were achieved in the duck cell lines pIX and CR, as well as HEK_Dyn_, HEK_FS_, and HEK_PEM_ (Figure 1g and Table 1). However, only low CSVYs up to 9 PFU/cell were reached.

This observation shows the profound effects of not only cell origin, but also cell line history and culture media on virus replication.

### 3.2. Accelerated Parallel Assessment of Critical Process Parameters

**Figure 2:**
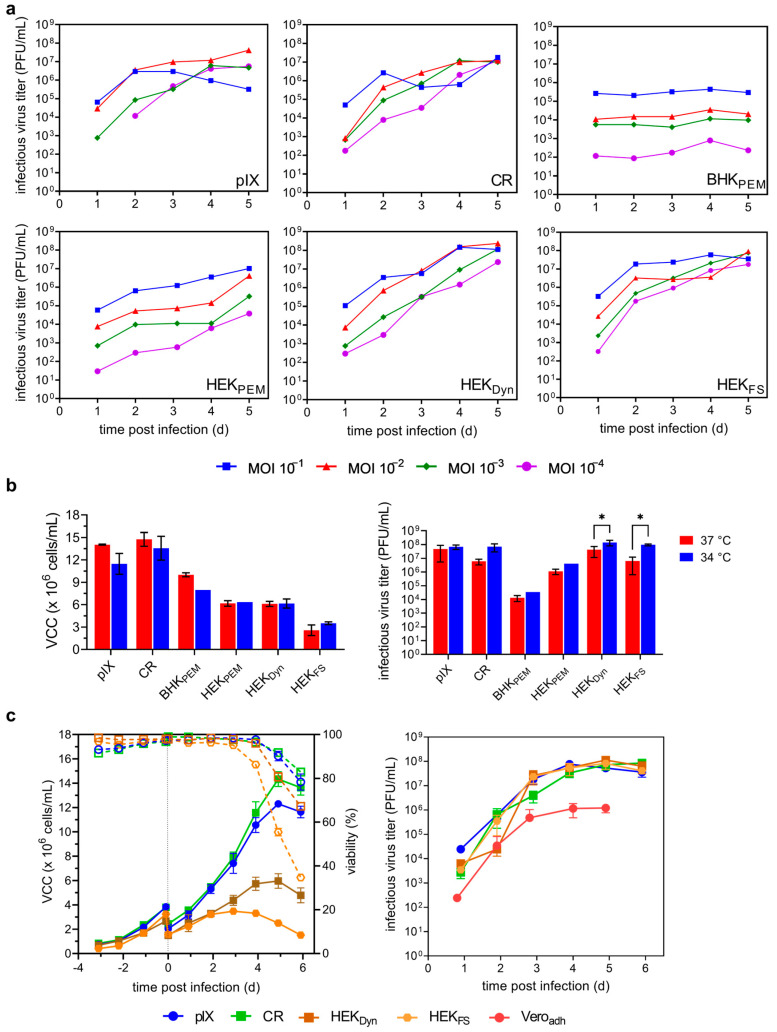
Optimizing YF-ZIK production in selected host cells. Effect of MOI and temperature on infectious virus titers in ambr15 system. (**a**) MOI screening. Cells infected at MOI 0.1 (blue squares), 0.01 (red triangles), 0.001 (green diamonds), and 10^−4^ (purple circles). Values from a single cultivation. (**b**) Impact of temperature on maximum viable cell concentrations (VCC_max_) post infection (left) and resulting infectious virus titers (right). Values mean ± STD of n = 2 for runs at 37 °C and n = 4 for runs at 34 °C. BHK_PEM_ and HEK_PEM_ at 34 °C as single runs. Two-way ANOVA followed by Šidák’s multiple comparison; *p* values * < 0.05 were considered significant. (**c**) Optimized productions were repeated as a biological triplicate to confirm reproducibility and robustness. VCCs (full symbols) and culture viabilities (empty symbols and dashed lines) are shown left, infectious virus titers right. Dashed lines indicate time of infection. Values are represented as the mean ± STD of n = 3.

Next, we investigated parameters relevant to productivity in a batch process, while minimizing additional process complexity. For this, we selected six host cell lines including all four high producers (pIX, CR, HEK_Dyn_, HEK_FS_), one low producer (HEK_PEM_), and one no producer (BHK_PEM_) under previous standard cultivation conditions. We assessed the effect of two process parameters (MOI and temperature) on infectious virus titers and confirmed the robustness of the resulting optimized process conditions in three biologically independent experiments (Figure 2). Cells were infected at MOIs ranging from 0.1 to 10^−4^ at 37 °C, or after reducing the temperature to 34 °C.

Higher amounts of virus input (MOI 0.1 or 0.01) resulted in higher (pIX, BHK_PEM_, HEK_PEM_, HEK_Dyn_) or similar infectious virus titers (CR, HEK_FS_) compared to lower virus inputs (MOI 10^−3^ or 10^−4^) (Figure 2a). Interestingly, no impact on VCC_max_ was observed for BHK_PEM_, HEK_PEM_, HEK_Dyn_, and HEK_FS_ cells regardless of virus input, while higher MOIs resulted in lower VCCs_max_ for both duck cell lines pIX and CR (Supplementary Table S4). Peak titers were reached between days 4 and 5 regardless of the initial virus input (except pIX at MOI 0.1; Figure 2a). However, each cell line clearly had a different optimal MOI. The highest maximum titers observed were 4.1 × 10^7^ PFU/mL at an MOI of 0.01 for pIX, 1.2 × 10^7^ PFU/mL at an MOI of 0.001 for CR, 4.4 × 10^5^ PFU/mL at an MOI of 0.1 for BHK_PEM_, 1.0 × 10^7^ PFU/mL at an MOI of 0.1 for HEK_PEM_, 2.4 × 10^8^ PFU/mL at an MOI of 0.01 for HEK_Dyn_, and 9.2 × 10^7^ PFU/mL at an MOI of 0.001 for HEK_FS_ (Figure 2a). Both HEK_Dyn_ and HEK_FS_ achieved strongly improved CSVYs of 35 and 19 PFU/cell, respectively (Supplementary Table S3). In the next step, the impact of reducing cultivation temperature to 34 °C on VCCs_max_ and infectious virus titers was evaluated using the optimal MOIs identified. While µ and VCC_max_ (Table 1 and Table 2) were generally lower at reduced temperatures for both duck cell lines, as well as for BHK_PEM_, this decrease was only significant for pIX cells (Figure 2b). There was no measurable effect on VCC_max_ or general growth kinetics (Table 1 and Table 2) for any HEK-derived cell line. Lowering the temperature to 34 °C at time of infection (TOI) resulted in higher infectious virus titers for all host cell lines; however, only for HEK_Dyn_ and HEK_FS_ were the 3.4-fold and 13.2-fold increases significant (*p* = 0.022 and *p* = 0.049; two-way ANOVA followed by Šidák’s multiple comparison test). As BHK_PEM_ and HEK_PEM_ remained either no or low producers, both were excluded from further investigations. The reproducibility and robustness of the identified optimizations were subsequently confirmed in three independent experiments (Figure 2c, Table 2).

In summary, the optimization of two critical parameters for virus production reliably increased infectious virus titers for all selected cell lines without increasing the complexity of the production process.

### 3.3. Fed-Batch Process with Early Infection at Low MOI

As YF-ZIK replicates slowly and VCCs continue to increase for up to 4–5 days p.i. (Figure 2c), we previously diluted the suspension culture 1.7–2-fold immediately prior to infection. However, because such a step complicates large-scale industrial batch productions, we omitted this initial cell expansion phase for HEK_Dyn_ and pIX cells and instead infected the cells just one day after seeding. Additionally, the previously identified optimum MOI (Table 2) was reduced by 1 log_10_ to further minimize the input of virus seed and to allow for additional cycles of viral amplification (now referred to as early low infection, ELI mode). Finally, the impact of daily addition of concentrated feeds (three different fed-batch modes, FB1–3) combined with the ELI mode on virus yields was evaluated. For pIX cells, FB3 was omitted, as the respective feed is specifically designed for HEK cells and has previously demonstrated poor performance in another avian cell line. Due to the fixed working volume requirements in the ambr15 systems, no metabolite measurements could be carried out.

**Figure 3:**
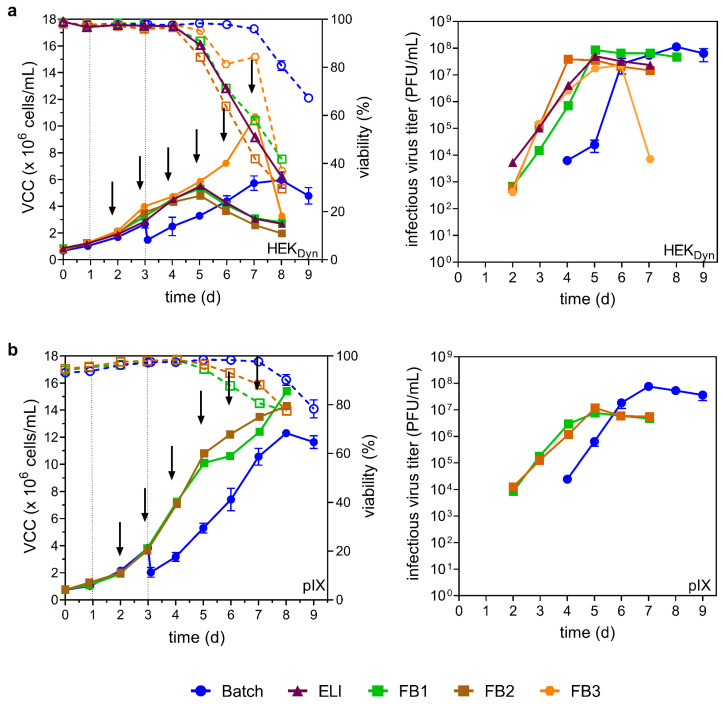
YF-ZIK production using HEK_Dyn_ (**a**) and pIX (**b**) cells in batch, ELI mode, and fed-batch mode. Values from batch mode (blue circles) from Figure 2c for comparison. VCCs (full symbols) and viability (empty symbols and dashed lines) are shown left, infectious virus titer right. For productions using early low infection mode (ELI, purple triangle) or fed-batch modes (FB1–3), cells were already infected one day after inoculation. Feeds used for fed-batch mode were media (FB1, green square), CHO Feed 1 (FB2, brown square), or HEKFS (FB3, orange circle). Arrows indicate the time of feeding; dashed lines indicate time of infection. Values from single experiments, except batch runs, are shown as mean ± STD with n = 3. Working volume increase due to feed: FB1 20%, FB2 18%, FB3 33%. Infectious virus titer and VCC are not corrected for volume increase.

As shown in Figure 3a, ELI mode, FB1 and FB2 did not result in higher VCCs compared to the batch process for HEK_Dyn_ cells. However, the maximum values were reached earlier (day 5 vs. day 8). For FB3, the cells continued to grow until day 7, reaching a VCC_max_ of 10.7 × 10^6^ cells/mL (1.8-fold increase compared to batch). Surprisingly, both ELI mode and fed-batch processes resulted in steep declines in cell viability after day 4 (3 days p.i.), with final culture viabilities below 40% (Figure 3a). FB3 resulted in the lowest maximum infectious virus titer of 2.4 × 10^7^ PFU/mL at day 6, followed by a drastic decrease of 3 log_10_ after culture viability decreased by 40%. The highest maximum titers observed were 5.0 × 10^7^ PFU/mL for ELI, 8.5 × 10^7^ PFU/mL for FB1, 3.8 × 10^7^ PFU/mL for FB2, and 2.4 × 10^7^ PFU/mL for FB3. While all process variations resulted in markedly lower maximum infectious virus titers compared to the batch control (about 1.3–4.8 fold; Table 2), maximal titers were reached 3–4 days earlier. However, both FB1 and FB2 supported cell growth to higher VCCs compared to the batch process (Figure 3b). Here, VCCs_max_ of 15.4 × 10^6^ cells/mL for FB1 and 14.3 × 10^6^ cells/mL for FB2 were reached on day 8. Infectious virus titers were not improved by FB1 or FB2; however, for HEK_Dyn_ cells, maximum infectious virus titers were reached earlier (day 5 compared to day 8 for the batch control).

Taken together, the combination of ELI mode and fed-batch operation resulted in significantly decreased production times; however, maximum infectious virus titers were reduced up to 4.8-fold when neglecting the volume increase by feeding.

### 3.4. Scale-Up to Laboratory Scale Stirred Tank Bioreactor

**Figure 4:**
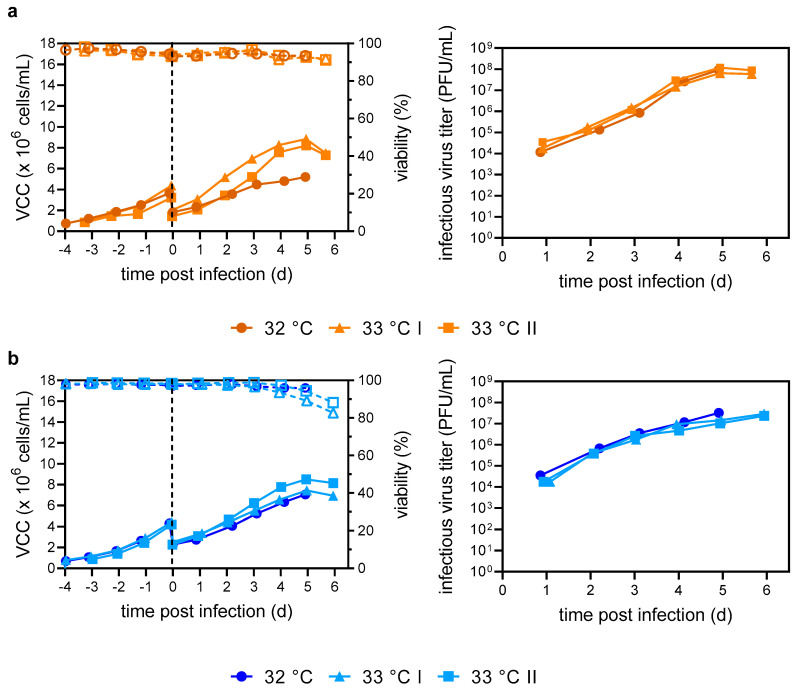
YF-ZIK production in batch mode using HEK_Dyn_ (**a**) and pIX cells (**b**) in three independent 1 L STR production runs at different infection temperatures. At time of infection, temperature was reduced to either 32 °C (circle) or 33 °C (triangle/square). VCCs (full symbols) and culture viabilities (empty symbols and dashed lines) shown left, infectious virus titers right. Dashed lines indicate time of infection. Values from single cultivations.

To demonstrate translation of the pH-, temperature-, agitation- and DO-controlled processes that had been optimized at a small scale (10^−2^ L vessels) directly for larger bioreactors, we carried out pilot runs in 1 L benchtop STRs. As temperature p.i. was identified as a significant factor for increased infectious virus titers in HEK_Dyn_ cells (Figure 2b), a further reduction to 32 and 33 °C was investigated. Both HEK_Dyn_ and pIX cells were seeded exactly as for the ambr15 system at 0.8 × 10^6^ cells/mL and cultivated until a VCC of about 4.0 × 10^6^ cells/mL was obtained (Figure 4a,b).

Here, cell-specific growth rates of 0.018 ± 0.001 1/h and 0.020 ± 0.002 1/h for HEK_Dyn_ and pIX cells were reached, which were consistent with previous cultivations in the ambr15 system (Table 1 and Table 2). After infection, HEK_Dyn_ cells continued to grow until 5 days p.i, with both reactors infected at 33 °C showing similar cell-specific growth rates of 0.014 ± 0.002 1/h and growth curves before the onset of cell lysis (VCC_max_ 8.2–8.4 × 10^6^ cells/mL). At 32 °C, µ decreased to 0.008 1/h, and a lower VCC_max_ of 5.2 × 10^6^ cells/mL was reached (Figure 4a). Despite differing growth kinetics at 32 and 33 °C, infectious virus titers were similar, reaching maximum titers of 0.7–1.2 × 10^8^ PFU/mL and CSVYs of 7–18 PFU/cell, as in the ambr15 system (Figure 4a; Table 1 and Table 2). For pIX cells, there was no discernible difference in cell-specific growth rate (0.009–0.011 1/h) nor VCC_max_ (7.1–8.2 × 10^6^ cells/mL) p.i., regardless of the temperature (Figure 4b). Interestingly, VCCs_max_ were 1.5-fold lower compared to the ambr15 system operated at 34 °C. Maximum infectious virus titers were comparable (1.5–3.2 × 10^7^ PFU/mL) across all conditions, but three times lower compared to ambr15 system cultivations (Figure 4b, Table 1 and Table 2). Nevertheless, due to the lower VCCs_max_, similar CSVYs of 3–5 PFU/cell were reached.

For HEK_FS_ and CR cells, only one run each at 32 °C was carried out (Supplementary Figure S1a). Here, maximum infectious virus titers of 6.5 × 10^7^ PFU/mL and 2.4 × 10^6^ PFU/mL were reached, respectively. Regardless of the cell line, there was no limitation in glucose or glutamine, nor an accumulation of secondary by-products to critical levels over the entire process time (Supplementary Figure S1b). Reactors operated at 32 °C were harvested at day 5 p.i., the cells were removed by centrifugation at 1500× *g* at 4 °C for 15 min, and the supernatant was stored at −80 °C.

### 3.5. In Vitro Characterization of STR YF-ZIK Batches from Different Host Cell Lines

**Figure 5:**
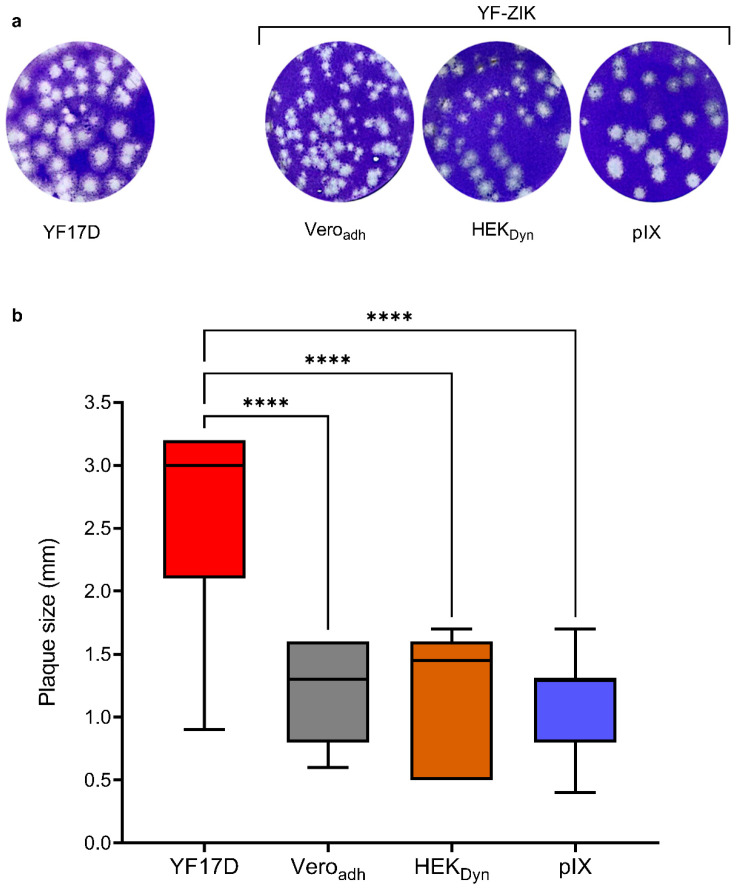
In vitro characterization of STR YF-ZIK batches produced in selected host cells. Plaque morphology of YF-ZIK batches vs. parental YF17D virus. BHK-21J cells in 6-well plates infected and plaques visualized 6 days post infection. (**a**) Plaque phenotypes of different YF-ZIK batches and YF17D are shown. (**b**) Size distribution of plaques. Median ± IQR for 100–150 individual plaques for each batch. Error bars represent the lowest and highest values. Kruskal–Wallis test followed by Dunn’s multiple comparison test, **** *p* < 0.0001.

A change of line and growth conditions may induce genetic adaptations in the produced viruses, which could potentially impact the consistency and safety of vaccine lots. Therefore, YF-ZIK produced in 1 L STRs at 32 °C from pIX, CR, HEK_Dyn_ and HEK_FS_ cells as well as the YF-ZIK stock produced in Vero_adh_ were selected for further in vitro and in vivo characterization. To obtain the full-length RNA genomic sequence for assessment of the genetic stability (identity and homogeneity) of the different YF-ZIK batches, five cDNA fragments (see Supplementary Table S3) covering the entire genome of YF-ZIK from each batch were amplified by RT-PCR, visualized and purified by agarose gel electrophoresis, and subjected to direct Sanger sequencing. A subsequent sequence comparison showed full identity with passage 1 Vero_adh_ YF-ZIK stock. To determine the accurate virus input for subsequent in vivo studies, the previously determined infectious virus titers were validated using a plaque assay on BHK-21J cells (Supplementary Table S5). Lastly, the plaque morphology of the different YF-ZIK batches was assessed and compared to parental YF17D (Figure 5a). As expected, the resulting plaque sizes of Vero_adh_-, HEK_Dyn_-, and pIX-derived YF-ZIK were homogenous, and remained all significantly reduced (*p* < 0.0001) compared to YF17D (Figure 5b) [12], suggesting preservation of an overall high homogeneity, and likewise no emergence of more aggressively growing live vaccine virus variants of general safety concern.

### 3.6. In Vivo Characterization of STR YF-ZIK Batches from Different Host Cell Lines

**Figure 6:**
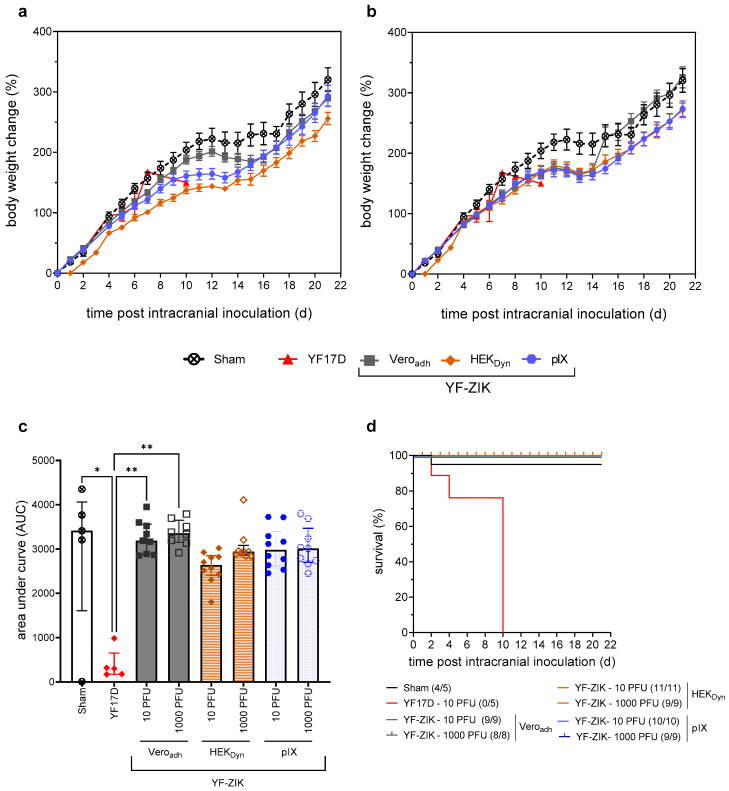
In vivo characterization of neurovirulence of STR YF-ZIK batches from different cell lines. Comparative analysis of BALB/c pups following i.c. inoculation of 10 (**a**) or 1000 PFU (**b**) of YF-ZIK derived from pIX (blue circles), HEK_Dyn_ (brown diamonds), and Vero_adh_ cells (gray squares), or 10 PFU of YF17D (red triangles) or sham (hollow circles). (**a**) Weight evolution of 3–6-day-old pups after i.c. with 10 PFU of YF-ZIK grown on Vero_adh_ (original seed virus; n = 9), HEK_Dyn_ (n = 11), or pIX cells (n = 10), 10 PFU of YF17D (positive control; n = 5) or sham. (**b**) Item using 1000 PFU of YF-ZIK. (**c**) Area under the curve (AUC) mean ± SEM. Kruskal–Wallis test followed by Dunn’s multiple comparison to assess the statistical significance with * *p* < 0.05 and ** *p* < 0.01 and (**d**) Survival rate (number of surviving mice at the endpoint is indicated).

YF-ZIK batches produced in pIX and HEK_Dyn_ cells at the 1L STR scale were selected for final assessment of neurovirulence and immunogenicity and compared to parental YF17D- and Vero_adh_-grown YF-ZIK, the starting seed material (Figure 6). Mirroring a classical YF17D neurovirulence/potency test [12], groups of suckling BALB/c pups (n = 8–11) were inoculated intracranially with 10 PFU or 1000 PFU of either Vero_adh_-, HEK_Dyn_-, or pIX-derived YF-ZIK, or 10 PFU of YF17D (n = 5) or medium (sham) as positive and negative controls, respectively. Intriguingly, even when using up to 100-fold higher input doses (1000 PFU) of YF-ZIK, all pups survived, in contrast to those inoculated with as little as 10 PFU of YF17D (Figure 6d), which is known for its pronounced neurovirulence in suckling mice (*p* <  0.0001, log-rank test). The body weight was compared between the treatment groups by calculation of the area under the curve (AUC, Figure 6c). There was no significant difference in body weight between the sham group and pups treated with 10 or 1000 PFU YF-ZIK, regardless of the host cell origin. Conversely, the AUC was significantly lower for pups inoculated with YF17D compared to the sham group (*p* = 0.018) as well as 10 and 1000 PFU of YF-ZIK-Vero_adh_ (*p* = 0.089 and *p* = 0.001). In summary, the production process (cell line, medium, and scale of production) did not affect the favorable safety profile of YF-ZIK, which appeared to be improved over the parental YF17D [12,30].

**Figure 7:**
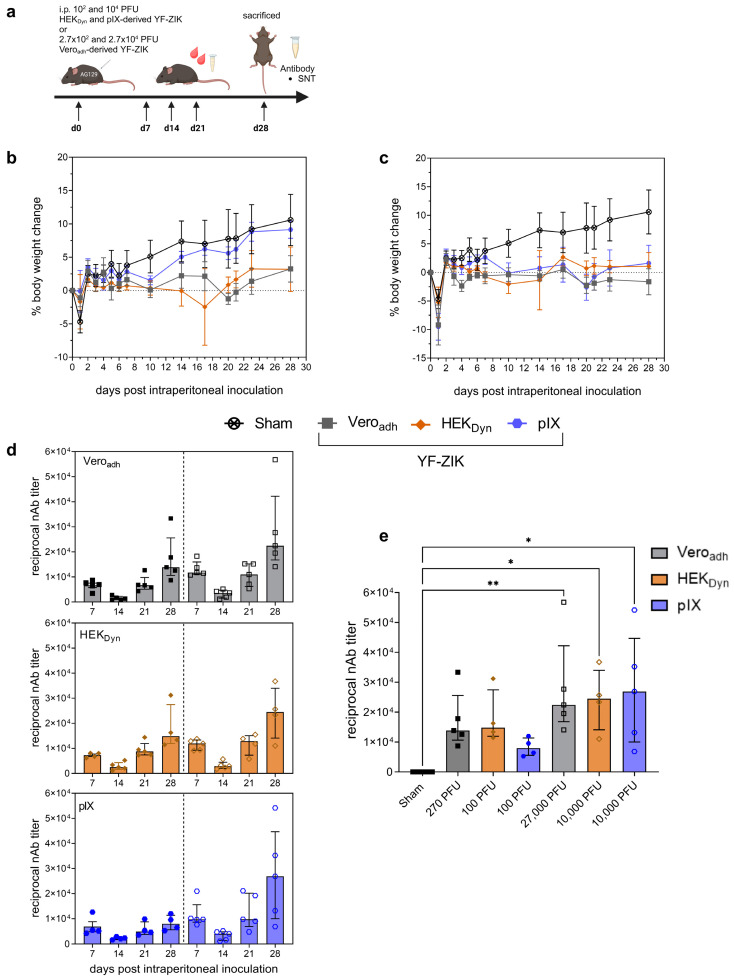
In vivo characterization of safety and immunogenicity of YF-ZIK from 1 L STR batches. (**a**) AG129 mice inoculated i.p. with high (10^4^ PFU) or low (10^2^ PFU) doses of YF-ZIK derived from HEK_Dyn_ and pIX cells; doses for Vero_adh_-derived YF-ZIK were about 2.7× higher. (**b**,**c**) Body weight evolution after vaccination with a low (**b**) or high dose (**c**) of Vero_ad_- (gray squares), HEK_Dyn_- (brown diamonds), and pIX-derived YF-ZIK (blue circles), or sham (asterisk). (**d**) Reciprocal nAb kinetics after vaccination for both low (full symbols) and high (hollow symbols) doses of Vero_adh_- (gray), HEK_Dyn_- (brown) and pIX (blue)-derived YF-ZIK. Low and high doses separated by dashed line. (**e**) Reciprocal nAb titers on day 28 p.i. after vaccination with YF-ZIK batches. Data mean ± SEM (**b**,**c**) or median ± IQR (**d**,**e**). Two-tailed Kruskal–Wallis test followed by Dunn’s multiple comparison, significant *p* values * < 0.05 and ** <0.01 as indicated.

To assess ZIKV-specific immune responses elicited by YF-ZIK produced on Vero_adh_, HEK_Dyn_, or pIX, and to exclude any neuroinvasive properties, we used adult IFN type I and II receptor-knockout (AG129 [21,22]) mice, which are highly susceptible to lethal orthoflavivirus infection. In line with anticipated vaccine safety and as previously demonstrated for Vero E60derived YF-ZIK [12], i.p. vaccination with YF-ZIK at any dose (Supplementary Table S6) did not result in any overt disease symptoms (normal behavior, coat, gait, and posture). The majority of AG129 mice did not exhibit body weight loss (Figure 7b,c). Only one out of five mice receiving either 100 or 10^4^ PFU of HEK_Dyn_-derived vaccine experienced a significant (>15%) body weight loss, requiring euthanasia. In contrast, YF17D was uniformly lethal in this model at doses as low < 1 PFU [12,31]. Animals vaccinated with a low 100 PFU dose of pIX-derived material showed a slight gain in body weight similar to sham-treated mice (Figure 7b).

Finally, all n  =  30 AG129 mice that were vaccinated with YF-ZIK seroconverted to high titers of ZIKV-neutralizing antibodies (nAb) as early as 7 days post vaccination (Figure 7d). Furthermore, the animals remained seropositive until the end of the study (day 28 p.i.), regardless of the host cell origin. This was observed to occur in a dose-dependent manner, though the results were not statistically significant when analyzed using the Kruskal–Wallis test (Figure 7d,e).

## 4. Discussion

Vaccination is a fundamental strategy for the prevention of endemic or emerging infectious diseases. Therefore, the implementation and development of vaccine technologies that allow for timely and scalable manufacture are highly desired [26]. Despite several advancements, the inherent biological complexity and resulting variability in the manufacturing process, coupled with strict regulatory specifications, add to the lengthy and costly nature of traditional vaccine production [32]. Novel platform solutions entailing multiple alternative approaches for antigen and vector design, production, and formulation [33,34] are required to match both the speed and productivity of emerging technologies such as mRNA as well as the need to adapt processes to yield drug products of highest desirable safety and immunogenicity [35,36]. Overall, it thus becomes evident that universally applicable manufacturing solutions do not exist. Here, we describe a workflow for upstream process development for the high-yield production of live recombinant YF17D-vectored vaccines using YF-ZIK as an example [12]. Our stepwise approach guides efficient process optimization by screening multiple parameters in a targeted high-throughput manner, providing a blueprint that might be applicable to other live-attenuated and vectored viral vaccines both established and currently in development.

### 4.1. Selection of a Favorable Cell Line

The type, culture format, and origin of the host cell have an enormous impact on the yield and quality of a vaccine production process [37,38,39]. In the current study, we started testing with a large panel of suspension cell lines as possible host cell lines for the YF-ZIK replication. Out of the 14 tested suspension cell lines, only 5 supported notable YF-ZIK production, with select human (HEK) and avian (CR, pIX) cell lines showing the most promising productivity (Table 1, Figure 1g). Intriguingly, besides MDCK (canine) cells that had not been tested before for YF-ZIK, BHK-21 (hamster) and Vero (simian) cells were poor producers. This is unexpected as the latter cells—yet originating from different sources and grown in adherent monolayers—had previously been shown to support infection with YF-ZIK [12] as well as with both YF17D and ZIKV [28,40]. Additionally, a similar YF17D derivative ChimeriVax-Zika, described by others [25], had been grown to high yields in Vero_adh_ cells (although it is difficult to compare absolute virus titers between studies) [12,25]. This failure to support productive infection (Figure 1g) was furthermore unexpected, because we obtained high titers for the parental YF17D in MDCK [41,42] and BHK-21 cells [28], whereas the YF-ZIK high producers (pIX, CR, and HEK cells) identified here showed only poor yields for the parental YF17D in a previous study [42]. In fact, infection of HEK_Dyn_ and pIX cells with YF-ZIK yielded over a 30-fold higher infectious virus titer than Vero_adh_ cells, even considering that CSVYs were initially low (Table 1). Our comprehensive parallel screening approach demonstrates the importance of a rapid cell line selection protocol at the onset of a vaccine production development, even for closely related vectored constructs. Compared to process development based on a single cell line, our approach is more likely to yield significant process improvements while simultaneously reducing the total time required for process development (Supplementary Figure S2).

### 4.2. Optimizing Culture Conditions

In addition to the culture format and media, reducing the temperature during the viral replication phase can increase virus stability and/or maximum virus titers [43,44,45,46,47]. Here, a reduction in temperature p.i. to 34 °C significantly increased infectious virus titers in HEK_Dyn_ and HEK_FS_ cells. In particular, for pIX and CR cells, the µ p.i. was decreased with the temperature, resulting in a prolonged cell growth phase (5 days vs. 3 days at 37 °C; Figure 1a, Figure 2c). While VCCs_max_ were not affected, viabilities at time of peak virus titer were higher. A greater proportion of intact cells limits the amount of host cell-derived debris and the release of protein (including proteases) and DNA. The combined benefits include lower levels of impurities at the onset of subsequent downstream operations, reduced loss of infectious units to adsorption to debris or by proteolytical digestion, and improved physical stability of particles at lower temperatures.

We also investigated the effect of MOI on YF-ZIK production. Varying the amount of virus input at the time of infection has been described to primarily influence viral replication kinetics [48,49], as higher MOIs lead to an instantly increased population of infected cells. Except for pIX cells, the maximum infectious virus titers were reached on day 5 p.i., irrespective of the virus input (Figure 2a). Lower MOIs (10^−2^ to 10^−3^) resulted in the highest infectious virus titers for all YF-ZIK-replicating cell lines (Supplementary Table S3). Although not investigated further in this study but possibly important in subsequent downstream developments, the fraction of infectious virions to the viral genome copies (I/NI) is an additional critical production parameter that is responsive to process temperatures and time to harvest [50]. Here, the optimized process temperature (<37 °C) and MOI (<10^−2^) revealed four potential producer cell lines (pIX, CR, HEK_Dyn_, and HEK_FS_ cells) with similar virus production kinetics and markedly higher yields, resulting in up to more than 100-fold higher peak titers compared to Vero_adh_ cells (Table 2).

### 4.3. Proof of Concept for Translatability and Scale-Up

Concerning the operating expenditures associated with virus production processes, the input of virus seed has been identified as a significant cost factor (e.g., up to 27% of total cost for modified vaccinia Ankara virus [51]). As the maximum infectious virus titers were previously not negatively affected by lowering the MOI (Figure 2), we implemented a multiple infection cycle/low-MOI approach to minimize the required virus input. We further combined this approach with (i) an early infection [35], 1 day post seeding, as used for, e.g., hepatitis C virus as another slow-replicating virus [52], and (ii) feeding strategies to prevent nutrient limitations (Figure 3). Although for HEK_Dyn_ cells, process variations resulted in lower viabilities and lower peak titers compared to the batch control (Table 2), the expansion of the infected culture by the addition of feeds (1.2–1.3-fold) yielded similar absolute amount of infectious virus particles and peak titers up to 3 days earlier with 10-fold reductions in requirements for virus seed (Figure 3a). As previously discussed, lower viabilities may complicate downstream processing and must be calculated against the savings made by more economical use of input virus.

Direct scale-up over three orders of magnitude can be challenging and usually requires thorough engineering considerations. In addition, the particular geometry and small height of the ambr15 vessels result in a distinct fluid dynamic profile and high specific power input [53]. Nevertheless, for virus production, three earlier studies have shown that scale-up or scale-down solely based on tip speed results in similar growth performance and production yields across scales (up to 3L) [54,55,56]. Here, we also show that comparable virus titers can be reached in the ambr15 vessel and the 1 L STR cultivations by using tip speed as the sole scale-up parameter. Further temperature reductions to 33 or 32 °C did not affect infectious virus titers; however, at 32 °C, final culture viabilities on day 5 p.i. were as high as 90% with obvious benefits regarding purification needs for a future drug substance (Figure 4).

### 4.4. Consistency of Live Vaccine Produced in an Intensified Process

The most important critical quality attribute of live vaccines is the maintenance of attenuation. Although the genetic stability of YF17D and its derivatives is high [31,57,58,59], a switch in cell line may lead to adaptive mutations that could impact virulence [60,61,62,63,64], vaccine safety, and potency [65,66,67,68]. Serial passaging of virus stocks has been shown to improve viral replication dynamics and titers of YF-ZIK in Vero_adh_ cells [12] and is commonly applied for other viruses [38,69]. It is noteworthy that YF-ZIK, which was originally derived from a molecular clone, only gained full replication competence and potency after several passages in Vero_adh._ This phenotype was acquired via critical amino acid changes in the E and Canch domains [12]. The earlier observation contrasts with our results, which demonstrate that YF-ZIK remained genetically stable in suspension cultures. For instance, serial passaging of YF-ZIK over five passages in BHK-21_PEM_ cells did not result in any obvious cell culture adaptation, but rather in virus extinction. More importantly, during enhanced replication in HEK_Dyn_ and pIX cells, Sanger sequencing did not reveal mutations in the YF-ZIK genome compared to the P1 Vero_adh_ YF-ZIK seed material. However, future studies that should include deep sequencing analysis of virus populations might be important to provide valuable insights into the genetic diversity, mutational landscape, and evolutionary dynamics of the virus [70].

Plaque phenotype analysis on adherent BHK-21J cells further supports that attenuation of YF-ZIK produced in pIX, CR, HEK_Dyn_, and HEK_FS_ cells was not affected. We found that plaques, regardless of the host cell origin and thus passaging history, remained uniform in appearance and significantly smaller in size than those formed by YF17D [12] (Figure 5b). This result suggests that there was no or very low variability within resulting virus populations without the formation of viral quasi-species [71], which is a favorable indication for the safety and immunogenicity of our vaccine candidate [31,68,72,73].

### 4.5. In Vivo Safety and Immunogenicity

Consistent with YF-ZIK produced in Vero_adh_ cells as well as other chimeric YF17D-based constructs [23,24,25,65], we observed that all YF-ZIK batches demonstrated an improved safety profile in vivo compared to parental YF17D. All tested batches of YF-ZIK were devoid of the high neurovirulence properties of YF17D when inoculated intracranially (even at very high doses) in BALB/c pups [65] (Figure 6a–d). The lack of neuroinvasive properties of YF-ZIK was further confirmed by the absence of neurotropic growth following systemic injection into highly susceptible (immunocompromised) AG129 mice [74] (Figure 7). Though active replication of the vaccine virus in AG129 mice had some impact on their weight gain, in total, only 2/10 mice exposed to YF-ZIK (produced in HEK_Dyn_ cells) suffered from significant weight loss until the humane endpoint.

In summary, we did not observe in orthogonal assays that the growth and massive amplification of YF-ZIK during replication in a new cell line and an intensified upstream process leads to any loss of attenuation. Conversely, the overall benign phenotype of YF-ZIK is preserved when tested in two highly sensitive in vivo models (BALB/c pups and AG129 mice), in which the parental YF17D strain is uniformly lethal.

Vaccine immunogenicity was also not affected. A single dose of both the low (pIX and HEK_Dyn_: 100 PFU or Vero_adh_: 270 PFU) and high dose (pIX and HEK_Dyn_: 1.0 × 10^4^ or Vero_adh_:2.7 × 10^4^ PFU) of YF-ZIK resulted in the rapid seroconversion (within 7 days) to high levels of ZIKV-specific binding antibodies (Figure 7c). As expected, nAb titers were significantly elevated following a 100-fold higher vaccine dose. However, importantly, no differences could be observed when comparing batches from different origins. This suggests that the 50–110-fold higher titers obtained in the intensified process translated directly into a linearly increased higher volumetric yield of active drug substance.

### 4.6. Implications of Improved Yields

Calculating with a similar downstream recovery of 40% as for inactivated YF vaccine [75], our processes would allow the production of approximately 3 × 10^5^ (pIX) to 5 × 10^5^ doses (HEK_Dyn_) per 1 L cultivation. Assuming 10^5^ PFU/dose for human application, such yields would significantly exceed the 1000 doses per liter that are required to be competitive from a manufacturing perspective [35]. To produce the same amount of doses in traditional systems, either 700–1200 specific pathogen-free eggs (400 doses/egg [75]) or 80 L of Vero_adh_ supernatant would be required.

## 5. Conclusions

All combined, we demonstrate a rapid, multifactorial, and highly parallel workflow with precisely regulated variations of culture conditions and infection parameters for identification of suitable host cells in different media. Starting at a small scale, we were able to identify two potential producer cell lines within a short timeframe (4–6 weeks), and to propose optimized conditions for scalable processes yielding over 100-fold increased virus titers compared to the current benchmark. Critical properties for vaccine candidates such as immunogenicity, attenuation, genetic, and phenotypic stability remained constant in both in vitro and in vivo studies. Such a workflow could serve as a roadmap to rapidly develop or optimize production processes for novel chimeric YF17D-based constructs and other live-attenuated vectored viral vaccines.

## Figures and Tables

**Table 1:** Growth characteristics of cell lines before and after infection and virus yields.

Cell Line	VCC_max_ p.i. (10^6^ Cells/mL)	µ (1/h)	µ_inf_ (1/h)	inf. vir. Titer (10^6^ PFU/mL)	CSVY (PFU/Cell)
Vero_PDM_	4.0 ± 0.4	0.02 ± 0.003	0.015	<0.01	<0.1
Vero_PDM/MDXK_	5.6 ± 0.4	0.022	0.014	<0.01	<0.1
pIX	14.1 ± 0.1	0.024	0.024	55.9 ± 29.1	4 ± 2
CR	14.8 ± 0.9	0.026 ± 0.002	0.021 ± 0.001	6.0 ± 2.7	<1
CCX.E10	5.7 ± 0.1	0.020	0.010	0.8 ± 0.1	<0.1
BHK_PEM_	10.0 ± 0.3	0.024 ± 0.002	0.017 ± 0.001	<0.01	<0.1
BHK_HIP_	12.4 ± 0.1	0.027	0.018	0.02	<0.1
BHK-P_PEM_	4.8 ± 0.3	0.024 ± 0.001	0.022 ± 0.001	<0.01	<0.1
HEK_PEM_	6.2 ± 0.4	0.021 ± 0.002	0.020 ± 0.001	1.1 ± 0.4	<1
HEK_FS_	2.6 ± 0.7	0.008	0.010 ± 0.002	10.6 ± 5.8	4 ± 2
HEK_Dyn_	6.1 ± 0.3	0.018	0.014	55.0 ± 13.7	9 ± 2
HEKvs	6.8 ± 0.4	0.017 ± 0.002	0.017 ± 0.002	0.3 ± 0.1	<0.1
PBG.PK-21	7.7 ± 0.5	0.014 ± 0.001	0.011	0.1	<0.1
MDCK_MDXK_	16.2 ± 0.3	0.035 ± 0.001	0.028	<0.01	<0.1
MDCK_DM_	8.1 ± 0.1	0.02 ± 0.002	0.017	<0.01	<0.1
MDCK_4C_	8.4 ± 0.3	0.023 ± 0.001	0.013	<0.01	<0.1
MDCK_S_	16.3 ± 0.2	0.031 ± 0.002	0.027	<0.01	<0.1
Vero_adh_	2.5 ± 0.1	0.028 ± 0.001	0.003 ± 0.001	1.5 ± 0.1	<1

Maximum viable cell concentration (VCC_max_) post infection (p.i.); cell-specific growth rate before (µ) and after infection (µ_inf_); maximum infectious virus titer (inf. vir. titer); cell-specific virus yield (CSVY). Orange: high producers; white: low producers; gray: no producers. All runs carried out in duplicates. Values mean ± STD. If no deviation is given, values are too small to be displayed.

**Table 2:** Growth and productivity of selected cell lines under optimized conditions.

Cell Line	MOI	VCC_max_ p.i. (10^6^ Cells/mL)	µ (1/h)	µ_inf_ (1/h)	inf. vir. Titer (10^7^ PFU/mL)	CSVY (PFU/Cell)
pIX	0.01	12.3 ± 0.2	0.022 ± 0.006	0.016 ± 0.006	7.7 ± 2.3	6 ± 2
CR	0.001	14.3 ± 0.6	0.021 ± 0.006	0.015 ± 0.004	8.5 ± 1.1	6 ± 1
HEK_Dyn_	0.01	6.0 ± 0.6	0.019 ± 0.004	0.013 ± 0.008	11.3 ± 1.2	19 ± 2
HEK_FS_	0.001	3.5 ± 0.2	0.028 ± 0.007	0.012 ± 0.007	8.3 ± 3.6	24 ± 11

Maximum viable cell concentration (VCC_max_) post infection (p.i.); growth rate before (µ) and after infection (µ_inf_); maximum infectious virus titer (inf. vir. titer); cell-specific virus yield (CSVY). Orange, high producers; white, low producers; gray, no producers. All runs carried out in duplicates. Values are given as the mean ± STD with n = 3.

## Data Availability

Data are available in the article and the Supplementary Materials. Additional data are available on request from the authors.

## Supplementary Materials

The following supporting information can be downloaded at: https://www.mdpi.com/article/10.3390/vaccines12070755/s1, Figure S1: Batch production of YF-ZIK in HEK_FS_ and CR cells in 1 L STR at 32 °C; Figure S2: Comparison between a single cell line (purple squares) or parallel multiple cell lines (green circles) screening approach at the onset of vaccine production development; Table S1: Overview of the screened host cell lines; Table S2: Cultivation parameters used in shake flasks, ambr15 and 1 L STR cultivations; Table S3: Primers utilized for RT-PCR amplification of cDNA fragments of each YF-ZIK batch and for sequencing of the YF-ZIK genome; Table S4: YF-ZIK infection of six suspension cell lines at different MOIs ranging from 10^−4^ to 10^−1^ in ambr15 vessels; Table S5: Infectious virus titers of YF-ZIK batches determined by plaque assay using BHK-21J cells to determine the dose for mice experiments; Table S6: Infectious virus titers back-titrated from the inocula used for neurotoxicity and immunogenicity mice studies.

## Abbreviations

µ	cell-specific growth rate
AUC	area under the curve
CSVY	cell-specific virus yield
CMC	carboxyl-methyl-cellulose
DO	dissolved oxygen
ELI	early low infection mode
hpi	hours post infection
i.p.	intraperitoneal
I/NI	ratio of infectious to non-infectious virions
JEV	Japanese encephalitis virus
MOI	multiplicity of infection
nAb	virus-neutralizing antibodies
PEM	protein expression medium
PFU	plaque-forming units
p.i.	post infection
PS	porcine kidney stable cells
RT-PCR	reverse-transcription chain reaction
SEM	standard error of the mean
STD	standard deviation
SNT	serum neutralizing titers
STR	stirred tank bioreactor
VCC	viable cell concentration
VCC_max_	maximum viable cell concentration
wv	working volume
YF17D	yellow fever vaccine strain 17D
YFV	yellow fever virus
YF-ZIK	chimeric YF-ZIKprM/E vaccine virus
ZIKV	Zika virus

## References

[1] Fauci A.S., Morens D.M. (2016). Zika Virus in the Americas—Yet Another Arbovirus Threat. N. Engl. J. Med..

[2] Musso D., Ko A.I., Baud D. (2019). Zika Virus Infection—After the Pandemic. N. Engl. J. Med..

[3] Yadav P.D., Kaur H., Gupta N., Sahay R.R., Sapkal G.N., Shete A.M., Deshpande G.R., Mohandas S., Majumdar T., Patil S. (2022). Zika a Vector Borne Disease Detected in Newer States of India Amidst the COVID-19 Pandemic. Front. Microbiol..

[4] Khongwichit S., Chuchaona W., Vongpunsawad S., Poovorawan Y. (2023). Molecular epidemiology, clinical analysis, and genetic characterization of Zika virus infections in Thailand (2020–2023). Sci. Rep..

[5] Plourde A.R., Bloch E.M. (2016). A Literature Review of Zika Virus. Emerg. Infect. Dis..

[6] Cao-Lormeau V.M., Blake A., Mons S., Lastère S., Roche C., Vanhomwegen J., Dub T., Baudouin L., Teissier A., Larre P. (2016). Guillain-Barré Syndrome outbreak associated with Zika virus infection in French Polynesia: A case-control study. Lancet.

[7] Lin H.H., Yip B.S., Huang L.M., Wu S.C. (2018). Zika virus structural biology and progress in vaccine development. Biotechnol. Adv..

[8] Han H.H., Diaz C., Acosta C.J., Liu M., Borkowski A. (2021). Safety and immunogenicity of a purified inactivated Zika virus vaccine candidate in healthy adults: An observer-blind, randomised, phase 1 trial. Lancet Infect. Dis..

[9] Medina L.O., To A., Lieberman M.M., Wong T.A.S., Namekar M., Nakano E., Andersen H., Yalley-Ogunro J., Greenhouse J., Higgs S. (2018). A Recombinant Subunit Based Zika Virus Vaccine Is Efficacious in Non-human Primates. Front. Immunol..

[10] Shan C., Muruato A.E., Nunes B.T.D., Luo H., Xie X., Medeiros D.B.A., Wakamiya M., Tesh R.B., Barrett A.D., Wang T. (2017). A live-attenuated Zika virus vaccine candidate induces sterilizing immunity in mouse models. Nat. Med..

[11] Abbink P., Stephenson K.E., Barouch D.H. (2018). Zika virus vaccines. Nat. Rev. Microbiol..

[12] Kum D.B., Mishra N., Boudewijns R., Gladwyn-Ng I., Alfano C., Ma J., Schmid M.A., Marques R.E., Schols D., Kaptein S. (2018). A yellow fever-Zika chimeric virus vaccine candidate protects against Zika infection and congenital malformations in mice. NPJ Vaccines.

[13] Wang Y., Ling L., Zhang Z., Marin-Lopez A. (2022). Current Advances in Zika Vaccine Development. Vaccines.

[14] Bloom K., van den Berg F., Arbuthnot P. (2021). Self-amplifying RNA vaccines for infectious diseases. Gene Ther..

[15] Zhang Y., Zeng G., Pan H., Li C., Hu Y., Chu K., Han W., Chen Z., Tang R., Yin W. (2021). Safety, tolerability, and immunogenicity of an inactivated SARS-CoV-2 vaccine in healthy adults aged 18–59 years: A randomised, double-blind, placebo-controlled, phase 1/2 clinical trial. Lancet Infect. Dis..

[16] Zhang G., Tang T., Chen Y., Huang X., Liang T. (2023). mRNA vaccines in disease prevention and treatment. Signal Transduct. Target. Ther..

[17] Theiler M., Smith H.H., Mortimer P. (2000). The use of yellow fever virus modified by in vitro cultivation for human immunization. Rev. Med. Virol..

[18] Seligman S.J. (2014). Risk groups for yellow fever vaccine-associated viscerotropic disease (YEL-AVD). Vaccine.

[19] Barrett A.D., Teuwen D.E. (2009). Yellow fever vaccine—How does it work and why do rare cases of serious adverse events take place?. Curr. Opin. Immunol..

[20] Bonaldo M.C., Sequeira P.C., Galler R. (2014). The yellow fever 17D virus as a platform for new live attenuated vaccines. Hum. Vaccines Immunother..

[21] Lemmens V., Kelchtermans L., Debaveye S., Chiu W., Vercruysse T., Ma J., Thibaut H.J., Neyts J., Sanchez-Felipe L., Dallmeier K. (2023). YF17D-vectored Ebola vaccine candidate protects mice against lethal surrogate Ebola and yellow fever virus challenge. npj Vaccines.

[22] Sanchez-Felipe L., Vercruysse T., Sharma S., Ma J., Lemmens V., Van Looveren D., Arkalagud Javarappa M.P., Boudewijns R., Malengier-Devlies B., Liesenborghs L. (2021). A single-dose live-attenuated YF17D-vectored SARS-CoV-2 vaccine candidate. Nature.

[23] Monath T.P., McCarthy K., Bedford P., Johnson C.T., Nichols R., Yoksan S., Marchesani R., Knauber M., Wells K.H., Arroyo J. (2002). Clinical proof of principle for ChimeriVax™: Recombinant live, attenuated vaccines against flavivirus infections. Vaccine.

[24] Arroyo J., Miller C., Catalan J., Myers G.A., Ratterree M.S., Trent D.W., Monath T.P. (2004). ChimeriVax-West Nile virus live-attenuated vaccine: Preclinical evaluation of safety, immunogenicity, and efficacy. J. Virol..

[25] Giel-Moloney M., Goncalvez A.P., Catalan J., Lecouturier V., Girerd-Chambaz Y., Diaz F., Maldonado-Arocho F., Gomila R.C., Bernard M.-C., Oomen R. (2018). Chimeric yellow fever 17D-Zika virus (ChimeriVax-Zika) as a live-attenuated Zika virus vaccine. Sci. Rep..

[26] Jones C.H., Jenkins M.P., Adam Williams B., Welch V.L., True J.M. (2024). Exploring the future adult vaccine landscape—Crowded schedules and new dynamics. npj Vaccines.

[27] Kum D.B., Boudewijns R., Ma J., Mishra N., Schols D., Neyts J., Dallmeier K. (2020). A chimeric yellow fever-Zika virus vaccine candidate fully protects against yellow fever virus infection in mice. Emerg. Microbes Infect..

[28] Nikolay A., Castilho L.R., Reichl U., Genzel Y. (2018). Propagation of Brazilian Zika virus strains in static and suspension cultures using Vero and BHK cells. Vaccine.

[29] Yang Y., Shan C., Zou J., Muruato A.E., Bruno D.N., de Almeida Medeiros Daniele B., Vasconcelos P.F.C., Rossi S.L., Weaver S.C., Xie X. (2017). A cDNA Clone-Launched Platform for High-Yield Production of Inactivated Zika Vaccine. EBioMedicine.

[30] May Fulton C., Bailey W.J. (2021). Live Viral Vaccine Neurovirulence Screening: Current and Future Models. Vaccines.

[31] Kum D.B., Mishra N., Vrancken B., Thibaut H.J., Wilder-Smith A., Lemey P., Neyts J., Dallmeier K. (2019). Limited evolution of the yellow fever virus 17d in a mouse infection model. Emerg. Microbes Infect..

[32] Ramin E., Cardillo A.G., Liebers R., Schmölder J., von Lieres E., Van Molle W., Niebel B., Natalis L., Meln I., Perea-Vélez M. (2024). Accelerating vaccine manufacturing development through model-based approaches: Current advances and future opportunities. Curr. Opin. Chem. Eng..

[33] Sallard E., Zhang W., Aydin M., Schröer K., Ehrhardt A. (2023). The Adenovirus Vector Platform: Novel Insights into Rational Vector Design and Lessons Learned from the COVID-19 Vaccine. Viruses.

[34] Coughlan L., Kremer E.J., Shayakhmetov D.M. (2022). Adenovirus-based vaccines—A platform for pandemic preparedness against emerging viral pathogens. Mol. Ther..

[35] Joe C.C.D., Segireddy R.R., Oliveira C., Berg A., Li Y., Doultsinos D., Scholze S., Ahmad A., Nestola P., Niemann J. (2024). Accelerated and intensified manufacturing of an adenovirus-vectored vaccine to enable rapid outbreak response. Biotechnol. Bioeng..

[36] Mendonça S.A., Lorincz R., Boucher P., Curiel D.T. (2021). Adenoviral vector vaccine platforms in the SARS-CoV-2 pandemic. npj Vaccines.

[37] Tahara M., Takeda M., Shirogane Y., Hashiguchi T., Ohno S., Yanagi Y. (2008). Measles virus infects both polarized epithelial and immune cells by using distinctive receptor-binding sites on its hemagglutinin. J. Virol..

[38] Göbel S., Kortum F., Chavez K.J., Jordan I., Sandig V., Reichl U., Altomonte J., Genzel Y. (2022). Cell-line screening and process development for a fusogenic oncolytic virus in small-scale suspension cultures. Appl. Microbiol. Biotechnol..

[39] Grein T.A., Schwebel F., Kress M., Loewe D., Dieken H., Salzig D., Weidner T., Czermak P. (2017). Screening different host cell lines for the dynamic production of measles virus. Biotechnol. Prog..

[40] Lindenbach B.D., Rice C.M. (1997). trans-Complementation of yellow fever virus NS1 reveals a role in early RNA replication. J. Virol..

[41] Park Y.W., Lee K.S., Lee B.-Y., Park M., Kim H., Kim Y.-H., Lee S.-J. (2015). Mdck-Derived Cell Lines Adapted to Serum-Free Culture and Suspension Culture and Method for Preparing Vaccine Virus Using the Cells. U.S. Patent.

[42] Nikolay A. (2020). Intensified Yellow Fever and Zika Virus Production in Animal Cell Culture.

[43] Wechuck J.B., Ozuer A., Goins W.F., Wolfe D., Oligino T., Glorioso J.C., Ataai M.M. (2002). Effect of temperature, medium composition, and cell passage on production of herpes-based viral vectors. Biotechnol. Bioeng..

[44] Kaptein L.C., Greijer A.E., Valerio D., van Beusechem V.W. (1997). Optimized conditions for the production of recombinant amphotropic retroviral vector preparations. Gene Ther..

[45] Petiot E., Jacob D., Lanthier S., Lohr V., Ansorge S., Kamen A.A. (2011). Metabolic and kinetic analyses of influenza production in perfusion HEK293 cell culture. BMC Biotechnol..

[46] Elahi S.M., Shen C.F., Gilbert R. (2019). Optimization of production of vesicular stomatitis virus (VSV) in suspension serum-free culture medium at high cell density. J. Biotechnol..

[47] Gélinas J.-F., Azizi H., Kiesslich S., Lanthier S., Perdersen J., Chahal P.S., Ansorge S., Kobinger G., Gilbert R., Kamen A.A. (2019). Production of rVSV-ZEBOV in serum-free suspension culture of HEK 293SF cells. Vaccine.

[48] Audsley J.M., Tannock G.A. (2005). The growth of attenuated influenza vaccine donor strains in continuous cell lines. J. Virol. Methods.

[49] Rüdiger D., Kupke S.Y., Laske T., Zmora P., Reichl U. (2019). Multiscale modeling of influenza A virus replication in cell cultures predicts infection dynamics for highly different infection conditions. PLoS Comput. Biol..

[50] Kiesslich S., Vila-Chã Losa J.P., Gélinas J.F., Kamen A.A. (2020). Serum-free production of rVSV-ZEBOV in Vero cells: Microcarrier bioreactor versus scale-X™ hydro fixed-bed. J. Biotechnol..

[51] Gränicher G., Babakhani M., Göbel S., Jordan I., Marichal-Gallardo P., Genzel Y., Reichl U. (2021). A high cell density perfusion process for Modified Vaccinia virus Ankara production: Process integration with inline DNA digestion and cost analysis. Biotechnol. Bioeng..

[52] Li Y.P., Ramirez S., Jensen S.B., Purcell R.H., Gottwein J.M., Bukh J. (2012). Highly efficient full-length hepatitis C virus genotype 1 (strain TN) infectious culture system. Proc. Natl. Acad. Sci. USA.

[53] Nienow A.W., Rielly C.D., Brosnan K., Bargh N., Lee K., Coopman K., Hewitt C.J. (2013). The physical characterisation of a microscale parallel bioreactor platform with an industrial CHO cell line expressing an IgG4. Biochem. Eng. J..

[54] Göbel S., Jaén K.E., Fernandes R.P., Reiter M., Altomonte J., Reichl U., Genzel Y. (2023). Characterization of a quail suspension cell line for production of a fusogenic oncolytic virus. Biotechnol. Bioeng..

[55] Chen P., Demirji J., Ivleva V.B., Horwitz J., Schwartz R., Arnold F. (2019). The transient expression of CHIKV VLP in large stirred tank bioreactors. Cytotechnology.

[56] Zinnecker T., Badri N., Araujo D., Thiele K., Reichl U., Genzel Y. (2024). From single-cell cloning to high-yield influenza virus production—Implementing advanced technologies in vaccine process development. Eng. Life Sci..

[57] Pugachev K.V., Guirakhoo F., Ocran S.W., Mitchell F., Parsons M., Penal C., Girakhoo S., Pougatcheva S.O., Arroyo J., Trent D.W. (2004). High fidelity of yellow fever virus RNA polymerase. J. Virol..

[58] Davis E.H., Beck A.S., Strother A.E., Thompson J.K., Widen S.G., Higgs S., Wood T.G., Barrett A.D.T. (2019). Attenuation of Live-Attenuated Yellow Fever 17D Vaccine Virus Is Localized to a High-Fidelity Replication Complex. mBio.

[59] Beasley D.W., Morin M., Lamb A.R., Hayman E., Watts D.M., Lee C.K., Trent D.W., Monath T.P. (2013). Adaptation of yellow fever virus 17D to Vero cells is associated with mutations in structural and non-structural protein genes. Virus Res..

[60] Converse J.L., Kovatch R.M., Pulliam J.D., Nagle S.C., Snyder E.M. (1971). Virulence and pathogenesis of yellow fever virus serially passaged in cell culture. Appl. Microbiol..

[61] Barrett A.D., Monath T.P., Cropp C.B., Adkins J.A., Ledger T.N., Gould E.A., Schlesinger J.J., Kinney R.M., Trent D.W. (1990). Attenuation of wild-type yellow fever virus by passage in HeLa cells. J. Gen. Virol..

[62] Ryman K.D., Xie H., Ledger T.N., Campbell G.A., Barrett A.D. (1997). Antigenic variants of yellow fever virus with an altered neurovirulence phenotype in mice. Virology.

[63] Ryman K.D., Ledger T.N., Campbell G.A., Watowich S.J., Barrett A.D. (1998). Mutation in a 17D-204 vaccine substrain-specific envelope protein epitope alters the pathogenesis of yellow fever virus in mice. Virology.

[64] Chambers T.J., Nickells M. (2001). Neuroadapted yellow fever virus 17D: Genetic and biological characterization of a highly mouse-neurovirulent virus and its infectious molecular clone. J. Virol..

[65] Monath T.P., Myers G.A., Beck R.A., Knauber M., Scappaticci K., Pullano T., Tad Archambault W., Catalan J., Miller C., Zhang Z.-X. (2005). Safety testing for neurovirulence of novel live, attenuated flavivirus vaccines: Infant mice provide an accurate surrogate for the test in monkeys. Biologicals.

[66] Guirakhoo F., Zhang Z., Myers G., Johnson B.W., Pugachev K., Nichols R., Brown N., Levenbook I., Draper K., Cyrek S. (2004). A single amino acid substitution in the envelope protein of chimeric yellow fever-dengue 1 vaccine virus reduces neurovirulence for suckling mice and viremia/viscerotropism for monkeys. J. Virol..

[67] Galler R., Pugachev K.V., Santos C.L., Ocran S.W., Jabor A.V., Rodrigues S.G., Marchevsky R.S., Freire M.S., Almeida L.F., Cruz A.C. (2001). Phenotypic and molecular analyses of yellow fever 17DD vaccine viruses associated with serious adverse events in Brazil. Virology.

[68] Esson R., Rodrigues De Sousa E., Benair L., Devard N., Soulet D., Gillet A., Bassard I., Falque S., Chareyre A., Marmin M. (2022). Phenotypic and genetic characterization of a next generation live-attenuated yellow fever vaccine candidate. Vaccine.

[69] Le Ru A., Jacob D., Transfiguracion J., Ansorge S., Henry O., Kamen A.A. (2010). Scalable production of influenza virus in HEK-293 cells for efficient vaccine manufacturing. Vaccine.

[70] Beck A., Tesh R.B., Wood T.G., Widen S.G., Ryman K.D., Barrett A.D. (2014). Comparison of the live attenuated yellow fever vaccine 17D-204 strain to its virulent parental strain Asibi by deep sequencing. J. Infect. Dis..

[71] Dangsagul W., Ruchusatsawat K., Tawatsin A., Changsom D., Noisumdaeng P., Putchakarn S., Phatihattakorn C., Auewarakul P., Puthavathana P. (2021). Zika virus isolation, propagation, and quantification using multiple methods. PLoS ONE.

[72] Barban V., Girerd Y., Aguirre M., Gulia S., Pétiard F., Riou P., Barrere B., Lang J. (2007). High stability of yellow fever 17D-204 vaccine: A 12-year restrospective analysis of large-scale production. Vaccine.

[73] Mantel N., Girerd Y., Geny C., Bernard I., Pontvianne J., Lang J., Barban V. (2011). Genetic stability of a dengue vaccine based on chimeric yellow fever/dengue viruses. Vaccine.

[74] Meier K.C., Gardner C.L., Khoretonenko M.V., Klimstra W.B., Ryman K.D. (2009). A mouse model for studying viscerotropic disease caused by yellow fever virus infection. PLoS Pathog..

[75] Pato T.P., Souza M.C.O., Mattos D.A., Caride E., Ferreira D.F., Gaspar L.P., Freire M.S., Castilho L.R. (2019). Purification of yellow fever virus produced in Vero cells for inactivated vaccine manufacture. Vaccine.

