# Supplementary material for: Parallel Multifactorial Process Optimization and Intensification for High-Yield Production of Live YF17D-Vectored Zika Vaccine
Source: Vaccines (Basel). 2024 Jul 9;12(7):755. doi: 10.3390/vaccines12070755 (PMC11281342; doi:10.3390/vaccines12070755)
Supplement: Supplementary file 1 [file vaccines-12-00755-s001.zip › vaccines-3036485-supplementary.pdf]

Journal: “Vaccines”

Manuscript Title: “Parallel Multifactorial Process Optimization and Intensification for High-Yield Production of Live YF17D-Vectored Zika Vaccine”

Supplementary Material

Table S1. Overview of the screened host cell lines.

	Cell line	Medium	Abbreviation	Supplement	Supplier
adherent	Vero WHO	GMEM-Z	$Vero_{adh}$	LaB-M-peptone + 10 % FCS	ECACC (88020401)
	Vero WHO	VPSFM	$Vero_{adh}$	4 mM Gln	ECACC (88020401)
	Vero E6	MEM	Vero E6	10 % FCS*, 2 mM Gln, 1 % $NAHCO_3$	LUMC (ATCC CRL-1586)
	BHK-21J	MEM	BHK-21J	10 % FCS*, 2 mM Gln, 1 % $NAHCO_3$	LUMC
	PS	GMEM-Z	PS	LaB-M-peptone + 10 % FCS	RKI
suspension	Vero	PDM	$Vero_{PDM}$	1/3 PEM + 1/3 CD-U7 + 1/3 Driving M + 9 mM Gln + 50 µL IGF*	MPI/UEL (ECACC (88020401))
	Vero	PDM/MDXK	$Vero_{PDM/MDXK}$	½ MDXK + ½ PCD + 1.5 mL AC a# + 1 mL AC b# + 1 mL growth factor#	MPI/UEL (ECACC (88020401))
	AGE1.CR.pIX	CD-U7	pIX	2 mM Gln, 2 mM Ala, 10 µL IGF*	PBG (Jordan et al 2019)
	AGE1.CR	CD-U7	CR	2 mM Gln, 2 mM Ala, 10 µL IGF*	PBG (Jordan et al 2019)
	CCX.E10	Freestyle	CCX.E10	growth factors	Nuvonis
	BHK-21	PEM	BHK_{PEM}	8 mM Gln, 4 mM Pyr	IDT Biologika/Ceva, In house (Nikolay 2014)
	BHK-21	HIP	BHK_{HIP}	8 mM Gln, 4 mM Pyr	In house
	BHK-21	PEM	BHK-P	8 mM Gln, 4 mM Pyr	PBG
	BHK-21	PEM	BHK-A	8 mM Gln, 4 mM Pyr	TUM (ATCC (C-13))
	HEK293	PEM	HEK_{PEM}	8 mM Gln, 4 mM Pyr	UAB/NRC
	HEK293-F	Freestyle	HEK_{FS}	-	ThermoFisher (R79007)
	HEK293-F	Dynamis	HEK_{Dyn}	-	In house (R79007)
	HEK293-vp	PEM	HEKvp	8 mM Gln, 4 mM Pyr	PBG
	PBG.PK-21	CD-U7	PBG.PK-21	2 mM Gln, 2 mM Ala, 10 µL IGF*	PBG

MDCK	MDXK	$MDCK_{MDXK}$	8 mM Gln	ECUST
MDCK	Driving M	$MDCK_{DM}$	8 mM Gln	ECACC (84121903)
MDCK	4Cell	$MDCK_{4C}$	-	ECACC (84121903)
MDCK	MDXK	$MDCK_{S}$	8 mM Gln	Sartorius (ATCC (CCL-34))

Medium: VPSFM, Freestyle^{TM} 293 expression medium (Freestyle), Glasgow minimum essential medium (GMEM), high-intensity perfusion medium (HIP), protein expression medium (PEM), and Dynamis^{TM}:ThermoFisher. Minimum essential medium (MEM): MEM Invitrogen. CD-U7: Probiogen. MDXK and 4Cell medium: Xell AG / Sartorius. Xeno-CDM (Driving M): Shanghai BioEngine Sci-Tech. **Supplements:** Gln = glutamine, FCS = fetal calf serum, Pyr = pyruvate, Ala = alanine. Unless otherwise indicated, all supplements were obtained from Sigma-Aldrich. [*]Long R^3 human insulin growth factor (IGF): Repligen . [#]AC supplement a, AC supplement b, and growth factor supplement (1005-0020): Xell AG. **Suppliers:** PBG = ProBioGen AG, ECUST= East China University of Science and Technology, UAB= Autonomous University of Barcelona, NRC= National Research Council of Canada, LUMC= Leids Universitair Medisch Centrum, RKI= Robert Koch-Institute, UEL= University of Applied Sciences Emden-Leer, TUM= Technical University of Munich.

Table S2. Cultivation parameters used in shake flasks, ambr15 and 1 L STR cultivations.

Cell line	SF type	rpm SF	rpm ambr15	tip speed ambr15 (m/s)	rpm STR	tip speed STR (m/s)
Vero	non-baffled	110	440	0.26	-	
AGE1.CR.pIX	baffled	185	800	0.47	180	0.47
AGE1.CR	baffled	185	800	0.47	180	0.47
CCX.E10	baffled	185	800	0.47	-	
BHK-21	baffled	185	800	0.47	-	
HEK293	baffled	125	800	0.47	180	0.47
HEK293-F	baffled	125	800	0.47	-	
HEK293-vp	baffled	125	800	0.47	-	
PBG.PK-21	baffled	185	440	0.26	-	
MDCK	non-baffled	120	440	0.26	-	

Tip speeds were calculated using a pitched blade diameter of 50 mm for the 1 L STR system and 11.4 mm for the ambr15 system. SF= shake flask, STR= stirred tank bioreactor

Table S3. Primers utilized for RT-PCR amplification of cDNA fragments of each YF-ZIK batch and for sequencing of the YF-ZIK genome.

Name	Sense	Sequence	
ChimZIK(+)1	Forward	AGTAAATCCTGTGTGCTA	Amplicon 1 2304 bp
ChimZIK(-)2304	Reverse	CCCAATGAGTTGAGAGCA	
ChimZIK(+)2205	Forward	AAGCATTTGAAGCCACT	Amplicon 2 2573 bp
ChimZIK(-)4778	Reverse	TGACATGCCACATTGTGT	
ChimZIK(+)4228	Forward	TATCCCAGTGAATGAGGCA	Amplicon 3 3078 bp
ChimZIK(-)7306	Reverse	AGGTAAAATGAGAGACCAGT	
ChimZIK(+)6784	Forward	ACCCACTCACATCTCCT	Amplicon 4 2492 bp
ChimZIK(-)9276	Reverse	TAGAATCCACCACCATCCAT	
ChimZIK(+)9056	Forward	AGAGAGAAGAAGCTGTCAGA	Amplicon 5 1851 bp
ChimZIK(-)10907	Reverse	AGTGGTTTTGTGTTTGTCATC	
ChimZIK(+)1	Forward	AGTAAATCCTGTGTGCTA	Sequencing
ChimZIK(+)120	Forward	TGTCTGGTCGTAAAGCTCA	
ChimZIK(+)169	Forward	CGTCAAGGAGAAAAAACTATAATGTC TGGTCGTAAAGCTC	
ChimZIK(+)208	Forward	AGTAAATCCTGTGTGCTAATTG	
ChimZIK(-)283	Reverse	AGCGTAATCTGGAACATCGTATGGG TAGCGAACTCCTCGTCGTAC	
ChimZIK(-)1804	Reverse	CCATGATCTGTATGTAACAC	
ChimZIK(+)1395	Forward	ATACTTGGTCATGATACTGCT	
ChimZIK(+)1396	Forward	GGGGTTTTGGAAGCCTAGGA	
ChimZIK(+)1807	Forward	GGCAAACTGTCGTGGT	
ChimZIK(+)1397	Forward	ACGCAGGGACAGATGGAC	
ChimZIK(+)1811	Forward	AAGCATTTGAAGCCACT	
ChimZIK(+)1813	Forward	GAGACACAGCCTGGGA	
ChimZIK(-)1809	Reverse	CCCAATGAGTTGAGAGCA	
ChimZIK(+)1818	Forward	GTTCTCCCCAGGGAGG	
ChimZIK(-)1812	Reverse	TTTCCATCTATGATGAAGCT	
YF-17D (+)1100	Forward	CACAATGCCGCCTGTGA	
YF-17D (+)1815	Forward	TATCCCAGTGAATGAGGCA	
YF-17D (-)1816	Reverse	TGACATGCCACATTGTGT	
YF-17D (+)344	Forward	AACAGGAACGGAGAGGTG	
YF-17D (+)333	Forward	AGGAAGATCATGAAAGTTG	
YF-17D (+)1042	Forward	GCCCCCACCAGGGTTGTTCTTTCT	
YF-17D (-)347	Reverse	CCATGACATTTGCAGCTC	

YF-17D (+)1823	Forward	ACCCACTCACATCTCCT
YF-17D (-)1104	Reverse	GGGGATCACAACCACCATCA
YF-17D (+)153	Forward	GCCTGGACAGTGTACGTTGG
YF-17D (-)1824	Reverse	AGGTAAAATGAGAGACCAGT
YF-17D (-)1825	Reverse	CACAGAGCAGAGGCATC
YF-17D (+)1827	Forward	TGGAAGAGACGGCCAT
YF-17D (-)2304	Reverse	CCCAATGAGTTGAGAGCA
YF-17D (+)1836	Forward	CCCTGTCCCATCTCAAC
YF-17D (-)1834	Reverse	CAATCATTCCACCCTTTTGATG
YF-17D (+)1839	Forward	TAGTCATCCATCGTATCCGA
YF-17D (-)1837	Reverse	TCAGATAAGCTCACCCAGT
YF-17D (+)161	Forward	GATATCAAAAACCTGGTTTCTGGGAC CTCCC
YF-17D (-)1840	Reverse	AGTGGTTTTGTGTTTGTCATC
YF-17D(-)555	Forward	AGTGGTTTTGTGTTTGTCATC

Table S4. YF-ZIK infection of six suspension cell lines at different MOIs ranging from 10^{-4} to 10^{-1} in ambr15 vessels.

Cell line	MOI	VCC_{max} p.i. (E6 cells/mL)	inf. vir. titer (E6 PFU/mL)	CSVY (PFU/cell)
pIX	1E-1	8.9	2.9	<1
pIX	1E-2	9.4	41.2	4
pIX	1E-3	12.0	6.2	1
pIX	1E-4	12.9	5.6	<1
CR	1E-1	9.2	17.6	2
CR	1E-2	11.3	12.1	1
CR	1E-3	8.0	11.8	2
CR	1E-4	13.3	11.8	1
BHK_{PEM}	1E-1	8.7	0.4	<0.1
BHK_{PEM}	1E-2	8.0	0.0	<0.1
BHK_{PEM}	1E-3	5.2	0.0	<0.1
BHK_{PEM}	1E-4	7.6	0.0	<0.1
HEK_{PEM}	1E-1	6.6	10.3	2
HEK_{PEM}	1E-2	6.4	4.1	1
HEK_{PEM}	1E-3	6.3	0.3	<0.1
HEK_{PEM}	1E-4	5.9	0.0	<0.1
HEK_{Dyn}	1E-1	5.8	147.1	26
HEK_{Dyn}	1E-2	6.7	235.3	35
HEK_{Dyn}	1E-3	6.6	117.6	19
HEK_{Dyn}	1E-4	6.7	23.5	4
HEK_{FS}	1E-1	3.4	58.8	18
HEK_{FS}	1E-2	3.6	73.0	20
HEK_{FS}	1E-3	3.8	73.5	19
HEK_{FS}	1E-4	3.4	17.6	5

The maximum viable cell concentration (VCC_{max}) post infection (p.i.), the maximum infectious virus titer in the supernatant, and the cell-specific virus yield (CSVY) of single runs were measured. MOI: multiplicity of infection (infectious virions/cell).

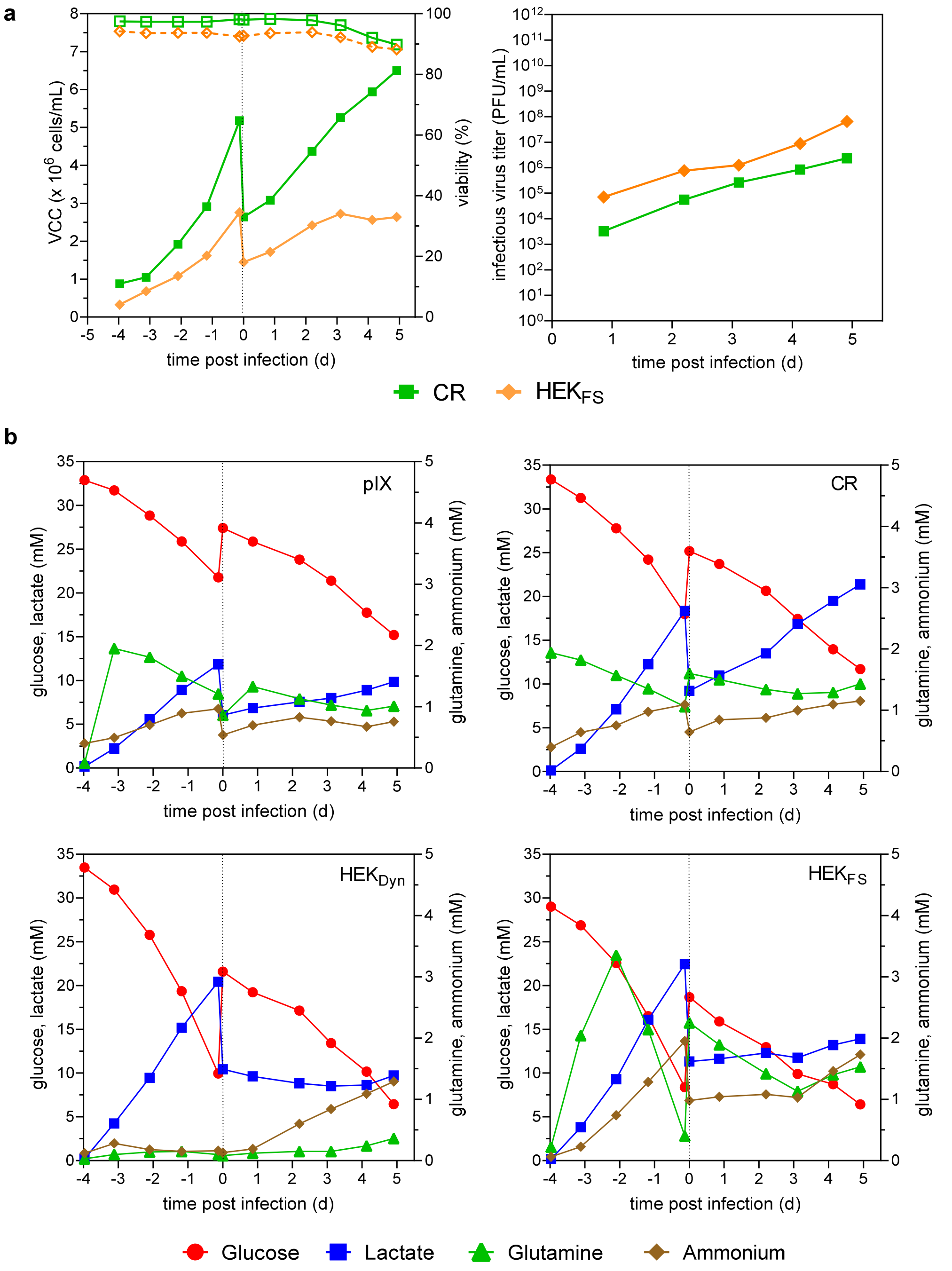

Figure S1. Batch production of YF-ZIK in HEK_{FS} and CR cells in 1 L STR at 32°C. HEK_{FS} and CR cells were grown in a 1 L STR (350 mL wv, 37°C). After an initial cell growth phase to about 4.0×10^6 cells/mL, cells were diluted with fresh medium (700 mL wv) and temperature was reduced to 32°C. Tip speeds from ambr 15 cultivations were used to determine stirring speeds for scale-up. a: Viable cell concentration (VCC), viability and infectious virus titers are shown for both CR and HEK_{FS} cells. b: Glucose (red circle), lactate (blue square), glutamine (green triangle), and ammonium (brown diamond) concentrations in cell culture supernatants were measured with a Cedex Bio Analyzer (Roche, Switzerland).

Table S5. Infectious virus titers of YF-ZIK batches determined by plaque assay using BHK-21J cells to determine the dose for mice experiments.

YF-ZIK batch	infectious virus titer (PFU/mL)
YF-ZIK-Vero$_{adh}$	$2.8x10^6$±1.5 $x10^6$
YF-ZIKV-HEK$_{Dyn}$	1.4 $x10^7$±5.4 $x10^6$
YF-ZIKV-HEK$_{FS}$	1.7 $x10^7$±4.9 $x10^6$
YF-ZIKV-pIX	9.5 $x10^6$±8.0 $x10^5$
YF-ZIKV-CR	4.2 $x10^5$±1.1 $x10^5$

Values are given as the mean±STD with n=3.

Table S6. Infectious virus titers back-titrated from the inocula used for neurotoxicity and immunogenicity mice studies.

Safety				
vaccine batch	high dose in log_{10} PFU		low dose in log_{10} PFU	
	intended injected dose	Back-titrated	intended injected dose	Back-titrated
YF-ZIK-$Vero_{adh}$	5	4.97	3	2.99
YF-ZIK-HEK_{Dyn}	5	4.94	3	2.94
YF-ZIK-pIX	5	4.7	3	3.02
YF17D	n.a.		3	3.09

Immunogenicity				
vaccine batch	high dose in log_{10} PFU		low dose in log_{10} PFU	
	intended injected dose	Back-titrated	intended injected dose	Back-titrated
YF-ZIK-$Vero_{adh}$	5.1	4.6	3.1	2.8
YF-ZIK-HEK_{Dyn}	4.7	4.7	2.7	2.2
YF-ZIK-pIX	4.7	4.0	2.7	2.4

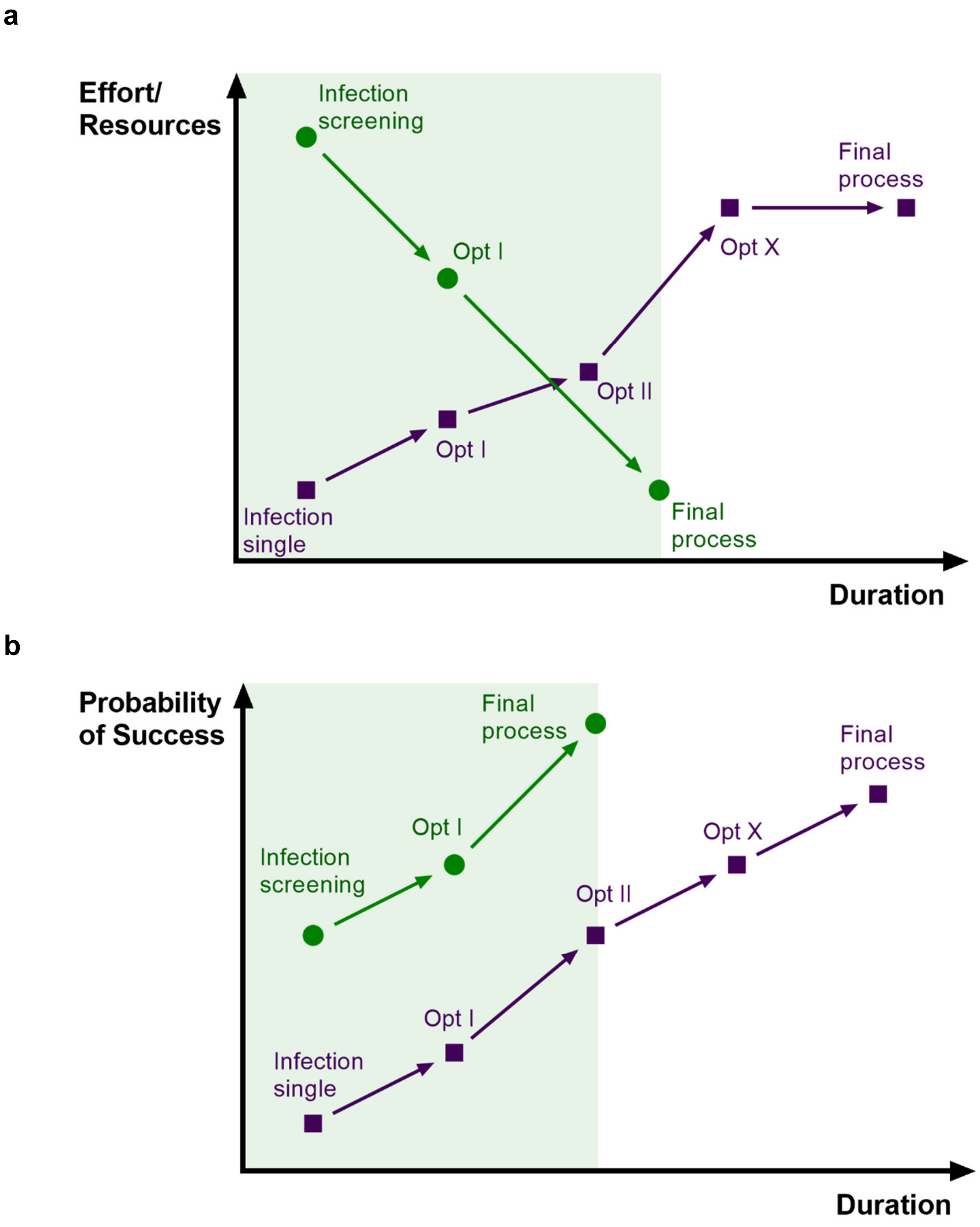

Figure S2. Comparison between a single cell line (purple squares) or parallel multiple cell lines (green circles) screening approach at the onset of vaccine production development. a: Comparison of effort/resource requirements over the time. **b:** Probability of success to achieve a significant process improvement over time.
